# A DOF transcriptional repressor-gibberellin feedback loop plays a crucial role in modulating light-independent seed germination

**DOI:** 10.1016/j.xplc.2025.101262

**Published:** 2025-01-28

**Authors:** Andrea Lepri, Hira Kazmi, Gaia Bertolotti, Chiara Longo, Sara Occhigrossi, Luca Quattrocchi, Mirko De Vivo, Daria Scintu, Noemi Svolacchia, Danuse Tarkowska, Veronika Tureckova, Miroslav Strnad, Marta Del Bianco, Riccardo Di Mambro, Paolo Costantino, Sabrina Sabatini, Raffaele Dello Ioio, Paola Vittorioso

**Affiliations:** 1 Department of Biology and Biotechnology Charles Darwin, University of Rome, Sapienza, P. le Aldo Moro 5, 00185 Rome, Italy; 2 Department of Biology and Biotechnology Charles Darwin, Sapienza, via dei Sardi, 70, 00185 Rome, Italy; 3 Laboratory of Growth Regulators, Institute of Experimental Botany, The Czech Academy of Sciences, & Faculty of Science, Palacky University Olomouc, Slechtitelu 27, CZ-78371 Olomouc, Czech Republic; 4 Italian Space Agency, Rome, Italy; 5 Department of Biology, University of Pisa, via L. Ghini, 13, 56126 Pisa, Italy

**Keywords:** seed germination, *Cardamine hirsuta*, DOF AFFECTING GERMINATION1, gibberellins, light

## Abstract

Plants have evolved several strategies to cope with the ever-changing environment. One example of this is given by seed germination, which must occur when environmental conditions are suitable for plant life. In the model system *Arabidopsis thaliana* seed germination is induced by light; however, in nature, seeds of several plant species can germinate regardless of this stimulus. While the molecular mechanisms underlying light-induced seed germination are well understood, those governing germination in the dark are still vague, mostly due to the lack of suitable model systems. Here, we employ *Cardamine hirsuta*, a close relative of *Arabidopsis*, as a powerful model system to uncover the molecular mechanisms underlying light-independent germination. By comparing *Cardamine* and *Arabidopsis*, we show that maintenance of the pro-germination hormone gibberellin (GA) levels prompt *Cardamine* seeds to germinate under both dark and light conditions. Using genetic and molecular biology experiments, we show that the *Cardamine* DOF transcriptional repressor DOF AFFECTING GERMINATION 1 (ChDAG1), homologous to the *Arabidopsis* transcription factor DAG1, is involved in this process functioning to mitigate GA levels by negatively regulating GA biosynthetic genes *ChGA3OX1 and ChGA3OX2*, independently of light conditions. We also demonstrate that this mechanism is likely conserved in other Brassicaceae species capable of germinating in dark conditions, such as *Lepidium sativum* and *Camelina sativa*. Our data support *Cardamine* as a new model system suitable for studying light-independent germination studies. Exploiting this system, we have also resolved a long-standing question about the mechanisms controlling light-independent germination in plants, opening new frontiers for future research.

## Teaser

Using *Cardamine hirsuta* as a model system of light-independent seed germination, this study provides evidence demonstrating that maintaining the levels of the pro-germination hormone gibberellin (GA) prompts *Cardamine* seeds to germinate under both dark and light conditions. The *Cardamine* DOF transcription factor DOF AFFECTING GERMINATION 1 (ChDAG1), homologous to AtDAG1, is involved in this process by reducing GA levels via repression of GA biosynthetic genes, independently of light conditions. This molecular mechanism is likely conserved in other Brassicaceae species that exhibit light-independent germination.

## Introduction

In the life cycle of plants, many developmental and growth processes are strictly dependent on light. One of these processes is seed germination, a process that must occur at the right time and in the right place. Indeed, to prevent vivipary or early germination and also promote seed dispersal, dormancy is established once seed maturation is completed (Bewley, 1997). Water, temperature, and light are among the environmental cues that most influence this process; of these, light can have divergent effects, depending on its intensity and wavelength, and on the plant species (Yang et al., 2020). Indeed, plant responsiveness to light for seed germination can vary depending mainly on their habitats. Seeds of some plants germinate regardless of the light conditions (light-independent). In contrast, others show light inhibition of germination (light-inhibited, negative photoblastic) or, conversely, germinate only in the presence of light (light-dependent, positive photoblastic) (Baskin and Baskin, 1998; Carta et al., 2017; Yang et al., 2020). The ecological significance of this difference is still debated. It might be that light inhibits germination in plants living in particular arid and hot conditions such as deserts or coasts (Górski and Górska, 1979; Carta et al., 2017), whereas the opposite depends on light-limited conditions and is typical of small-seeded plants, like *Arabidopsis thaliana* and *Lactuca sativa* (Borthwick et al., 1952, 1954; Shropshire et al., 1961). Indeed, the importance of light as a germination cue decreases in species with relatively large seeds, suggesting that light requirement and seed size might have coevolved (Milberg et al., 2000).

Several pieces of evidence about light-dependent seed germination and its fine-tuned control mechanisms (Tognacca and Botto, 2021) derive from *Arabidopsis thaliana*. In this species, the photoreceptors phytochromes (PHY) are required to trigger seed germination, with phytochrome B (phyB) playing a major role in the process. Indeed, during seed imbibition, phyB mediates the Red/Far Red (R/FR) Low Fluence Response (LFR) to induce seed germination, with the active form (phyB Pfr) promoting seed germination (Shinomura et al., 1994, 1998; Botto et al., 1996; Casal et al., 1998). phyB promotes seed germination also by controlling both gibberellin (GA) sensitivity and biosynthesis (Hilhorst and Karssen, 1988; Derkx et al., 1993; Yang et al., 1995; Yamaguchi et al., 1998; Ogawa et al., 2003), with GA representing the hormonal cue triggering germination. Besides phyB, phyE also contributes to light-mediated seed germination, as it is responsible for the R/FR-reversible induction of germination in the *phyAphyB* double mutant, and for the promotion of GA sensitivity in the absence of phyB (Hennig et al., 2002; Arana et al., 2014). Light, through phyB, also controls the levels of abscisic acid (ABA), the hormone which has an antagonistic function to GA, as it promotes dormancy while inhibiting germination. Indeed, phyB controls the GA/ABA ratio inducing expression of the *Arabidopsis* GA biosynthetic genes *GIBBERELLIN 3-OXIDASE 1* (*AtGA3OX1*) and *GIBBERELLIN 3-OXIDASE 2* (*AtGA3OX2*) (Yamaguchi et al., 1998; Seo et al., 2006; Toyomasu et al., 2008), while downregulating the expression of the main GA catabolic gene *AtGA2OX2* (Nakaminami et al., 2003; Oh et al., 2006; Yamauchi et al., 2007). On the other hand, ABA levels decrease after R light treatment (Toyomasu et al., 1994; Seo et al., 2006; Oh et al., 2007) due to the reduced expression level of the ABA biosynthetic genes and to the increase of the catabolic one, *AtCYP707A2* (Seo et al., 2006; Oh et al., 2007; Sawada et al., 2008). Given that this germination process is triggered by light, a mechanism to repress germination in the dark has evolved, with the bHLH transcription factor PIF1 (PHYTOCHROME INTERACTING FACTOR 1) playing a pivotal role as master repressor (Oh et al., 2004). In seeds imbibed in the dark, PIF1 directly activates transcription of the GA signaling negative regulators GIBBERELLIC ACID INSENSITIVE (GAI) and REPRESSOR OF GA (RGA) encoding genes (Oh et al., 2007). PIF1 also promotes the expression of downstream repressors, as DOF AFFECTING GERMINATION 1 (AtDAG1), which in turn regulates GA and ABA metabolism (Papi et al., 2000; Gabriele et al., 2010). AtDAG1 directly represses the GA biosynthetic gene *AtGA3OX1* and the ABA catabolic one *AtCYP707A2*, thus controlling the hormonal balance between GA and ABA during seed dormancy and germination (Gabriele et al., 2010; Boccaccini et al., 2016). Consistently, *Atdag1* mutant seeds have lower ABA levels in dry seeds and higher GA levels in imbibed seeds, in agreement with the overexpression of *AtGA3OX1* and *AtCYP707A2* (Boccaccini et al., 2016). Similarly, inactivation of *AtGAI* leads to overexpression of *AtGA3OX1* and, consistently, interaction with GAI is necessary for DAG1-mediated repression of *AtGA3OX1* (Boccaccini et al., 2014).

Although the molecular mechanisms underlying the light-dependent germination process have been thoroughly studied (Yang et al., 2020; Longo et al., 2021; Sajeev et al., 2024), how the light-independent germination process is controlled is still unclear. A close relative of *Arabidopsis*, *Lepidium sativum*, is known to exhibit light-independent seed germination (Morris et al., 2011) but, despite many physiological studies, the molecular mechanisms underlying this process have not yet been elucidated, as *Lepidium* is not genetically tractable. The comparative study of two accessions of *Aethionema arabicum*, Cyprus (CYP) and Turkey (TUR), showing light-inhibited and light-independent germination, respectively, revealed that the GA/ABA ratio is crucial for light control of germination and that several germination regulators in *Arabidopsis* seem to be involved in this process also in *Aethionema*, even if their expression profile does not allow to clearly unveil their role (Mérai et al., 2019). To gain further insights about seed germination in this species, it has been necessary to create a fast neutrons mutant collection to screen for mutants in light-mediated germination (Mérai et al., 2023). In this study, we first explore the germination properties of different genetically tractable close relatives of *Arabidopsis*. Then, among the species able to germinate both in light and dark conditions, we exploit the model system *Cardamine hirsuta* to unveil the molecular mechanisms underlying the light-independent seed germination process. Thanks to its genetic tractability, *Cardamine* has emerged as a powerful tool for comparative studies on leaf morphogenesis, and root, flower, and fruit patterning, and development and natural variation (Hay and Tsiantis, 2006; Hay et al., 2014; Vlad et al., 2014; Gan et al., 2016; Hofhuis et al., 2016; Di Ruocco et al., 2018; Baumgarten et al., 2023). Utilizing this model system, we show that *Cardamine* is also suitable for comparative germination studies. We demonstrate that levels of GA do not decrease in dark conditions with light-independent germination, as *Cardamine* seeds maintain constant promotion of GA biosynthetic gene expression during imbibition. We show that a negative feedback loop between GA and the dark germination repressor ChDAG1 is key for fine-tuning these levels, enabling germination in dark conditions. We also provide evidence that these mechanisms are likely to be utilized by other Brassica models to germinate in a light-independent manner such as *Lepidium* and *Camelina*. Overall, our results highlight the conserved molecular mechanisms that govern the light-independent germination of seeds.

## Results

### *Cardamine hirsuta* is a powerful model system for studying light-independent germination

**Figure 1:**
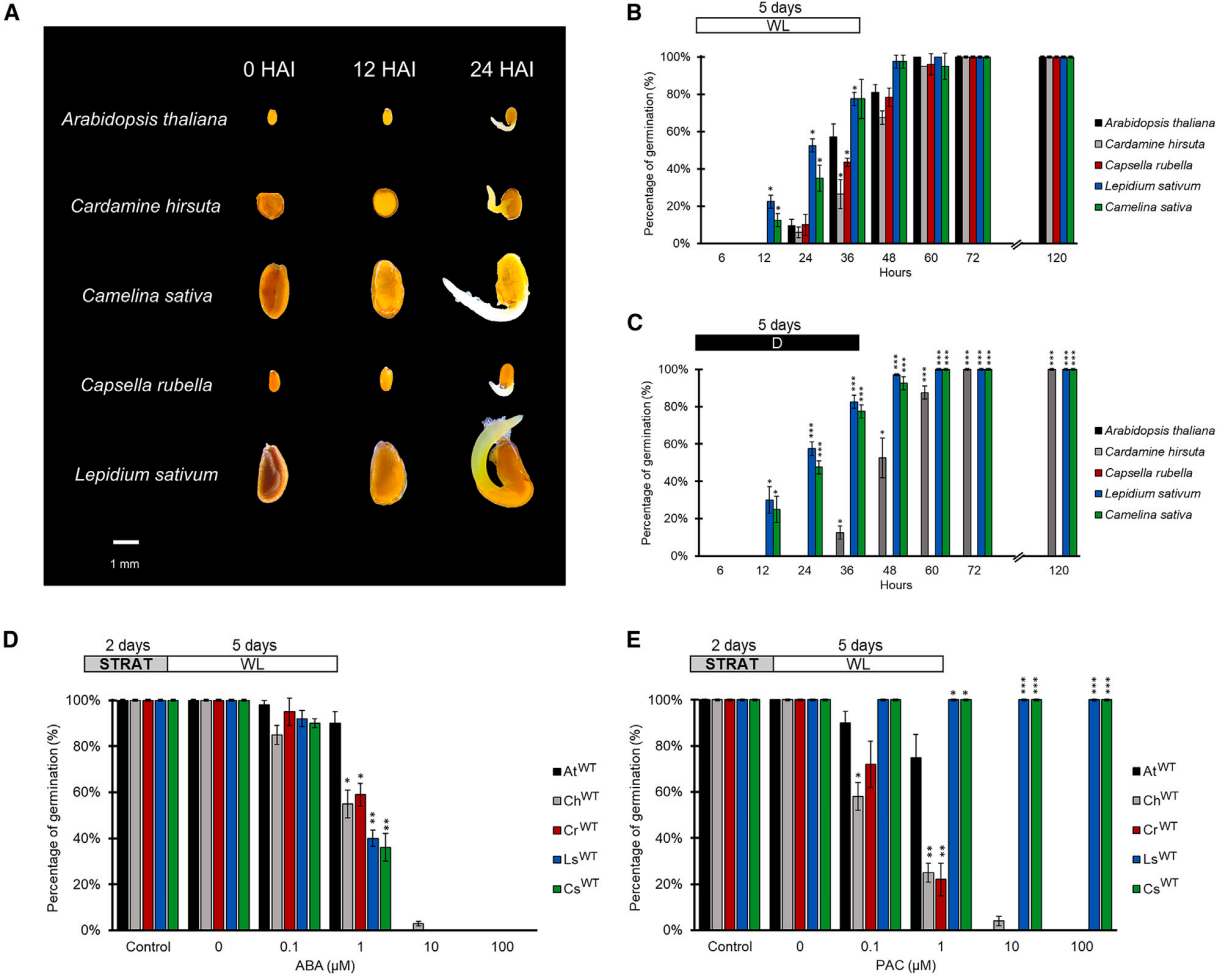
Light and hormonal requirements for the germination of *Cardamine*, *Capsella*, *Lepidium* and *Camelina* seeds. **(A)** Seed germination of *Cardamine hirsuta*, *Capsella rubella*, *Lepidium sativum* and *Camelina sativa* seeds, and *Arabidopsis thaliana* as a control from 0 to 24 HAI. **(B–E)** Germination rates: in white light **(B)**, total darkness **(C)**, with increasing ABA concentrations **(D)**, or PAC concentrations **(E)**. Germination rate was measured at different HAI (6, 12, 24, 36, 48, 60, 72 and 120) in **(B** **and C)**, and at 120 HAI in **(D** **and E)**. The values are means of three biological replicates, with SD values. Significant differences were analyzed by *t*-test (∗∗∗*p* ≤ 0.001, ∗∗*p* ≤ 0.005, ∗*p* ≤ 0.05). PAC, paclobutrazol; HAI, hours after imbibition. Control is referred to “mock treatment control” with ethanol. The diagram on top depicts the light treatment scheme; STRAT, stratification (2 days at 4°C, dark), WL, white light, D, dark.

Some information on seed germination derives from the plant model system *Arabidopsis*, nonetheless the germination properties and the molecular mechanisms underpinning these are likely to be different also in short evolutionary timescale. To identify novel molecular mechanisms governing light dependency for seed germination, we decided to isolate feasible model systems for comparative studies. To this end we analyzed the germination properties of genetically tractable close relatives of *Arabidopsis thaliana* and *Lepidium sativum*, such as *Cardamine hirsuta*, *Capsella rubella* and *Camelina sativa*. Comparative studies in closely related species have emerged as a powerful strategy to identify molecular mechanisms governing phenotypic differences. Hence, we characterize and compare the seed germination properties of these Brassica species with respect to light and hormone requirement. To assess the germination rate, we first monitored the kinetics of seed germination, where the percentage of germinating seeds was scored every 6–12 hours (h). Interestingly, *Lepidium* and *Camelina* seeds showed a faster germination rate, with 23% and 13% germinating seeds respectively, after 12 Hours After Imbibition (HAI) in white light. Conversely, *Cardamine* and *Capsella* seeds displayed a slower germination trend with 27% and 44% at 36 HAI, respectively, compared to 57% of *Arabidopsis* seeds (Figure 1A and 1B).

Seed germination in *Arabidopsis* and most annuals is induced by light, although for Brassicaceae such as *Lepidium sativum* and *Aethionema arabicum* light is not required or even inhibits germination (Shinomura et al., 1994; Mérai et al., 2019). Thus, we evaluated the light requirement for germination of *Cardamine*, *Camelina*and *Capsella* seeds and compared them with *Arabidopsis* and *Lepidium* seeds, as controls of light-dependent and light-independent germination. Seeds were sown under green, safe light and kept in total darkness up to 120 HAI. Germination frequency was measured every 6–12 h. Similar to the germination behavior under white light, *Lepidium* and *Camelina* showed 83% and 78% germinated seeds at 36 HAI, compared to 13% of *Cardamine*, although at 60 HAI the three species were almost fully germinated, while *Capsella* seeds did not germinate in the dark, as is the case with *Arabidopsis* seeds (Figure 1C).

GA and ABA levels are controlled by light in phyB-dependent seed germination: bioactive GAs increase after a R light pulse, while the amount of ABA decreases (Oh et al., 2006; Seo et al., 2006). Therefore, we wondered whether seed germination of the species showing light-independent germination process—namely *Lepidium*, *Cardamine* and *Camelina*—depended on these two hormone classes as is the case with *Arabidopsis* seeds. We thus performed a dose-response germination assay in the presence of increasing concentrations of ABA or paclobutrazol (PAC), an inhibitor of endogenous GA biosynthesis. Wild-type seeds of *Lepidium*, *Cardamine*, *Camelina*, and *Capsella* were more sensitive to the inhibitory effect of exogenous ABA compared to *Arabidopsis* seeds, as germination was significantly reduced at 1 μM ABA (Figure 1D). Unexpectedly, *Lepidium* and *Camelina* seeds were strikingly resistant to PAC, and showed 100% germination up to a 100 μM PAC concentration, whereas *Cardamine* and *Capsella* seeds were unable to germinate in the presence of 10 μM PAC, like *Arabidopsis* seeds (Figure 1E). By increasing PAC concentration up to 1 mM, germination of both *Lepidium* and *Camelina* seeds significantly decreased to 50% and 35%, respectively, combined with slower growth and poorly developed seedlings at 120 HAI, dropping down to 0 at 10 mM PAC (supplemental Figures 1A–1D).

This suggests that, despite the light independence for germination, the ratio between GAs and ABA levels is crucial for *Lepidium*, *Cardamine* and *Camelina* germination.

Given the availability of the genome sequence, as well as of a successful transformation method and short life cycle (Hay et al., 2014; Gan et al., 2016), we decided to focus our attention on *Cardamine* to gain insights in the light-independent germination process at both physiological and molecular level.

Since a reduced dormancy can result in increased germination potential, we assessed dormancy rate of *Cardamine* wild-type seeds, before going further on the study of seed germination in *Cardamine*. To this end, we measured germination frequency of freshly harvested seeds, and of seeds stored 1–4 weeks. Similarly to *Arabidopsis* seeds, freshly harvested *Cardamine* seeds were unable to germinate, while germination rate significantly increased during storage, reaching 100% after 4 weeks (supplemental Figure 2A). To further test the idea that *Cardamine* germination is light-independent, we investigated whether the process was therefore independent of phyB activity, by measuring germination frequencies of seeds exposed to a R or FR light pulse (5 minutes): the first converts phyB in the Pfr active form, while the second converts it in the Pr inactive form. We assessed the germination after the R-FR and R-FR-R treatments since once phyB is active, a FR pulse is able to switch it off to the Pr form, while a following R pulse converts it back to phyB Pfr (Borthwick et al., 1954; Shropshire et al., 1961). *Arabidopsis phyA* and *phyB* mutant seeds (Reed et al., 1993, 1994) with the corresponding wild-type (Col-0), were used as control. Remarkably, *Cardamine* seeds were able to germinate completely under all the light conditions tested, whereas the *Arabidopsis* wild-type seeds did not germinate after a FR pulse. The *Arabidopsis phyA* mutant seeds were unable to germinate after a FR pulse, while *phyB* could not germinate after the R pulse (supplemental Figure 2B). Hence, we identified *Cardamine* as a suitable model system to study the molecular basis of light-independent germination (supplemental Figure 3).

### GA levels are central to light-independent germination in *Cardamine hirsuta*

In *Arabidopsis*, GA and ABA levels are controlled by light during phyB-dependent seed germination, through the action of several downstream effectors (Oh et al., 2006; Seo et al., 2006). Indeed, the GA biosynthetic genes *AtGA3OX1* and *AtGA3OX2* are repressed in the dark, whereas the catabolic gene *AtGA2OX2* is induced. In contrast, in the same conditions the expression of the ABA biosynthetic genes, *AtABA1*, *AtNCED6*, and *AtNCED9*, is promoted, while the catabolic *AtCYP707A2* gene is downregulated (Oh et al., 2006; Seo et al., 2006). Given the light-independent germination of *Cardamine* seeds, we wondered whether the GA and ABA metabolic genes could have a different trend of expression in seeds imbibed in the dark.

**Figure 2:**
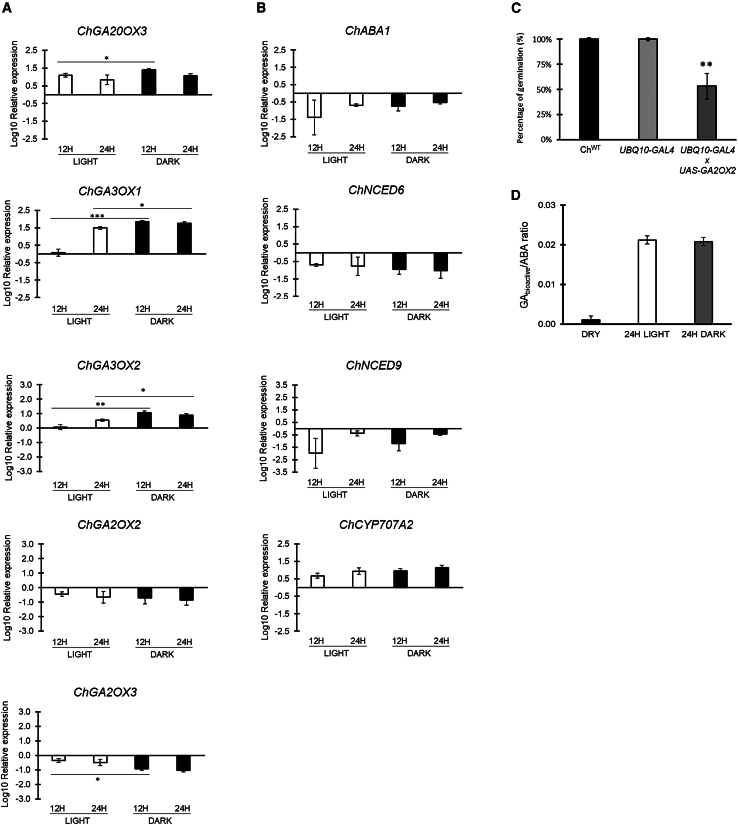
A high GA/ABA ratio enables the germination of *Cardamine* seeds in darkness. **(A and B)** Relative expression level of: *ChGA20OX3*, *ChGA3OX1*, *ChGA3OX2*, *ChGA2OX2*, *ChGA2OX3***(A)**, *ChABA1*, *ChNCED6*, *ChNCED9*, *ChCYP707A2***(B)** in *Cardamine* wild-type (Ox) seeds at 12 and 24 HAI (Hours After Imbibition), under light and dark conditions. Expression levels as log10 respect to the dry condition, set to 0 (X axis). The values are means of three biological replicates, with SD values. Significant differences were analyzed by *t*-test (∗∗∗*p* ≤ 0.001, ∗∗*p* ≤ 0.005, ∗*p* ≤ 0.05). **(C)** Germination of seeds *ChUBQ10>>GA2OX2*, issued from the cross *UBQ10::GAL4* × *UAS::GA2OX2*, under dark conditions, compared with the wild-type (Ox) and the *UBQ10-GAL4* line. Germination rates were measured at 120 HAI (Hours After Imbibition). The values are means of three biological replicates, with SD values. Significant differences were analyzed by one-way ANOVA with post hoc Tukey multiple comparison test (∗∗*p* ≤ 0.005). **(D)** Ratio of bioactive GAs/ABA in wild-type Ox seeds; the analyses were performed on dry and 24-h imbibed seeds in light and dark conditions. The values are the mean of three biological replicates, with SD values.

Since it is not yet known which GA and ABA metabolic genes are expressed in *Cardamine* seeds, we assessed the expression profiles of a number of both GA and ABA genes (supplemental Figure 4), on wild-type dry seeds and on seeds imbibed 12 or 24 h, either in white light or in total darkness, via real-time quantitative PCR (qPCR). The relative expression levels were compared to the dry condition, which was set to 0. The results of this analysis revealed that the GA and ABA metabolic genes mainly involved in seed germination are those reported in *Arabidopsis* (*ChGA20OX3*, *ChGA3OX1*, *ChGA3OX2*, *ChGA2OX2*, and *ChGA2OX3* for GA, *ChABA1*, *ChNCED6*, *ChNCED9* , and *ChCYP707A2* for ABA) with the sole exception of the catabolic gene *ChGA2OX3*, which in *Arabidopsis* is not expressed (https://www.arabidopsis.org/), while the others were very low or not expressed (Figure 2A and 2B and supplemental Figure 5).

Interestingly, expression of the GA biosynthetic gene *ChGA20OX3* was high irrespective of light conditions, while *ChGA3OX1* and *ChGA3OX2* were significantly upregulated during imbibition in the dark, compared to light conditions, where expression increased only at 24 HAI. Conversely, the mRNA levels of the catabolic genes *ChGA2OX2* and *ChGA2OX3* were low, both in light and dark conditions, compared to dry seeds (Figure 2A). In addition, expression of ABA genes showed an opposite trend, with the catabolic gene *ChCYP707A2* significantly upregulated during imbibition irrespective of the light conditions, while the biosynthetic genes were downregulated with respect to dry seeds (Figure 2B). These data suggest that the main regulators for the GA/ABA ratio have similar trends of expression in both light and dark conditions during imbibition, with best performances in dark. The expression profile of the GA metabolic genes in *Cardamine* was conserved in *Lepidium*, which is capable to germinate in a light-independent fashion, but not in *Capsella*, which requires light to germinate similarly to *Arabidopsis*, supporting a role for GA synthesis in the dark (supplemental Figure 6).

Given the expression profile of the GA biosynthetic and catabolic genes, we reasoned that overexpression of *ChGA2OX2*, encoding a GA-2 oxidase (Thomas et al., 1999), would lower GA levels in seeds and, in turn, possibly reduce the germination ability in the dark. To verify this hypothesis, we crossed the *Cardamine* transgenic lines expressing the transcriptional activator GAL4 under the control of the constitutive promoter *UBIQUITIN10* (*ChUBQ10::GAL4*), with lines bearing the *UAS::GA2OX2* construct (*ChUBQ10>>GA2OX2*) (Bertolotti et al., 2021). Germination in the dark of seeds issued from these crosses was significantly reduced in *ChUBQ10>>GA2OX2* in comparison with the *UBQ10-GAL4* control line and the wild-type (48.9% vs. 100%) (Figure 2C), corroborating the idea that control of GA levels is required to enable *Cardamine* to germinate in the dark.

As our data support the idea that GA levels must be increased during seed imbibition for consenting germination in dark conditions, we measured GA levels in dry seeds of *Cardamine*, as well as in 24-h imbibed seeds under dark or light conditions (supplemental Figure 7A). Given that the GA/ABA ratio influences seed germination, rather than the absolute levels of GAs, we also assessed ABA levels under the same conditions (supplemental Figure 7B). Consistent with the ability to germinate in the dark, GA levels in seeds imbibed in the dark were similar to those in light-imbibed seeds, as well as the amount of ABA in light-imbibed seeds was comparable with that of dark-imbibed seeds. This evidence suggests that light does not influence the metabolism of these phytohormones as it does in *Arabidopsis* seeds (Seo et al., 2006). Interestingly, and in contrast to dry seeds, the ratio of GA/ABA, based on the average of bioactive GAs (GA_1_, GA_4_, GA_7_, GA_5_, and GA_6_), was surprisingly high in both dark- and light-imbibed seeds (Figure 2D).

Our data suggest that increased levels of GAs during imbibition are key for triggering the germination of *Cardamine* seeds in a light-independent fashion.

### *ChDAG1* affects germination via fine-tuning GA levels during imbibition

**Figure 3:**
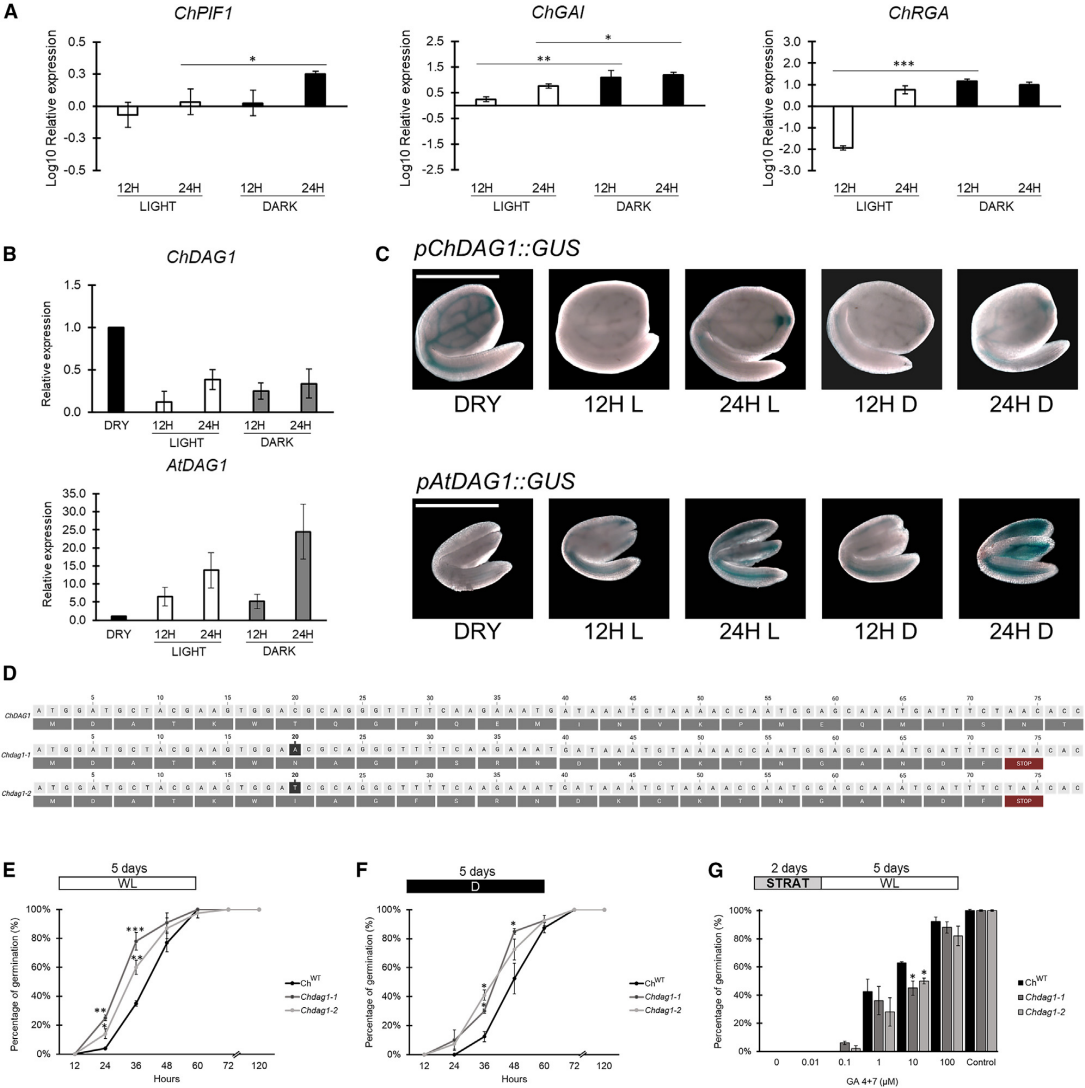
ChDAG1 is involved in light-independent seed germination. **(A and B)** Relative expression level of: *ChPIF1*, *ChGAI*, and *ChRGA***(A)**, *ChDAG1*and *AtDAG1***(B)** in *Cardamine* and *Arabidopsis* wild-type (Ox, Ws, respectively) seeds at 12 and 24 HAI (Hours After Imbibition), under light and dark conditions. Expression levels as log10 respect to the dry condition, set to 0 (X axis). The values are means of three biological replicates, with SD values. Significant differences were analyzed by *t*-test (∗∗∗*p* ≤ 0.001, ∗∗*p* ≤ 0.005, ∗*p* ≤ 0.05). **(C)** Histochemical staining of *pChDAG1::GUS* and *pAtDAG1::GUS* seeds dry (DRY) or imbibed 24 h, under white light (WL) or in dark (D). Scale bar, 1 mm. **(D)** Sequence of the *Chdag1-1* and *Chdag1-2* mutant alleles. **(E–G)** Germination rates of wild-type (Ox) and both *Chdag1-1* and *Chdag1-2* mutant seeds: in white light **(E)**, in total darkness **(F)**, and in the presence of PAC 100 μM + increasing concentrations of GAs **(G)**. Germination rates were measured at different HAI (12, 24, 36, 48, 60, 72, and 120) in **(E** **and F)**, and at 120 HAI in **(G)**. The values are means of three biological replicates, with SD values. Significant differences were analyzed by *t*-test (∗∗∗*p* ≤ 0.001, ∗∗*p* ≤ 0.005, ∗*p* ≤ 0.05). PAC, paclobutrazol; HAI, hours after imbibition. Control is referred to “mock treatment control” with ethanol. The diagram on top depicts the light treatment scheme; STRAT, stratification (2 days at 4°C, dark), WL, white light; D, dark.

In *Arabidopsis*, inhibition of dark germination occurs through the transcriptional activity of the master repressor AtPIF1, which positively controls downstream negative regulators of GA signaling and metabolism such as the DELLA proteins AtGAI and AtRGA and the DOF transcription factor AtDAG1 (Oh et al., 2004, 2007; Gabriele et al., 2010). Interestingly *ChPIF1* showed a low expression level during imbibition of *Cardamine* seeds, except at 24 HAI in the dark, when *ChPIF1* transcript level was significantly increased compared to 24 HAI in the light (Figure 3A), similarly to the *Arabidopsis AtPIF1* gene (https://bar.utoronto.ca/eplant). With respect to the expression profiles of the *DELLA* genes *ChGAI* and *ChRGA*, the transcript level of both genes was significantly higher during imbibition in the dark than in light conditions (Figure 3A). However, in contrast to *Arabidopsis* seeds, where *AtDAG1* expression level increases during dark-imbibition (Gabriele et al., 2010), *ChDAG1* expression was maintained low in both light- and dark-imbibed seeds, with respect to dry seeds (Figure 3B), suggesting a light-independent regulation for this transcription factor.

Given the role of DAG1 in the control of the GA/ABA ratio during seed germination in *Arabidopsis* (Boccaccini et al., 2016), we investigated the role of ChDAG1 in light-independent seed germination of *Cardamine*.

We first analyzed the expression of *ChDAG1* in *Cardamine* seeds during germination via the generation of *pChDAG1::GUS Cardamine* transgenic line, where the *GUS* reporter gene is under the control of 2.15 kb of the *ChDAG1* promoter region. Although this gene is expressed in the vascular tissue of the hypocotyl and cotyledons, similarly to *Arabidopsis*, the expression profile during germination is quite diverse, as shown by both the GUS assay and the real-time qPCR analysis. Noticeably, *ChDAG1* expression is higher in dry seeds than in dark- and light-imbibed seeds, whereas *AtDAG1* expression, as expected, is induced during imbibition, mainly in darkness (Figure 3B and 3C) (Gabriele et al., 2010).

By using the CRISPR-Cas9 methodology (Alvim Kamei et al., 2020), we generated a loss-of-function *Chdag1* mutant, designing a guide RNA (gRNA) immediately after the start codon of the *ChDAG1* locus. Two independent *dag1* mutant alleles (*Chdag1-1* and *Chdag1-2*) were isolated and characterized by the insertion of a single base in the seventh codon starting from the ATG, A in the *Chdag1-1* allele and T in the *Chdag1-2*, resulting in a frameshift and the formation of a premature stop codon (Figure 3D). A seed germination assay revealed that inactivation of *ChDAG1* results in faster germination kinetics, as 25% of *Chdag1-1* seeds and 14% of *Chdag1-2* seeds germinated at 24 HAI and 78% and 60% at 36 HAI, compared to 4% and 35% of wild-type seeds, respectively (Figure 3E). Even in dark germination, inactivation of *ChDAG1* results in faster germination compared to wild-type (10% and 8%, and 30% and 41% germination rate for the two alleles at 24 and 36 HAI, compared to 0% and 13% of the wild-type, respectively) (Figure 3F). Since the lack of *AtDAG1* in *Arabidopsis* seeds results in a reduced requirement of GAs to germinate (Gualberti et al., 2002), and considering that germination of *Cardamine* seeds requires GAs, we investigated whether inactivation of *ChDAG1* could reduce the GA requirement also in *Cardamine*. For this, we performed a germination assay in the presence of an inhibitory amount of PAC (100 μM) and increasing concentrations of exogenous GAs. Conversely to the phenotype of *Atdag1* seeds (Gualberti et al., 2002), inactivation of *ChDAG1* results in a slight but significant decrease of germination at 10 μM GA, as *Chdag1-1* and *Chdag1-2* seeds showed 45% germination rate compared to 63% of wild-type seeds (Figure 3G), suggesting a slightly different control of the GA/ABA ratio by DAG1 between the two species.

**Figure 4:**
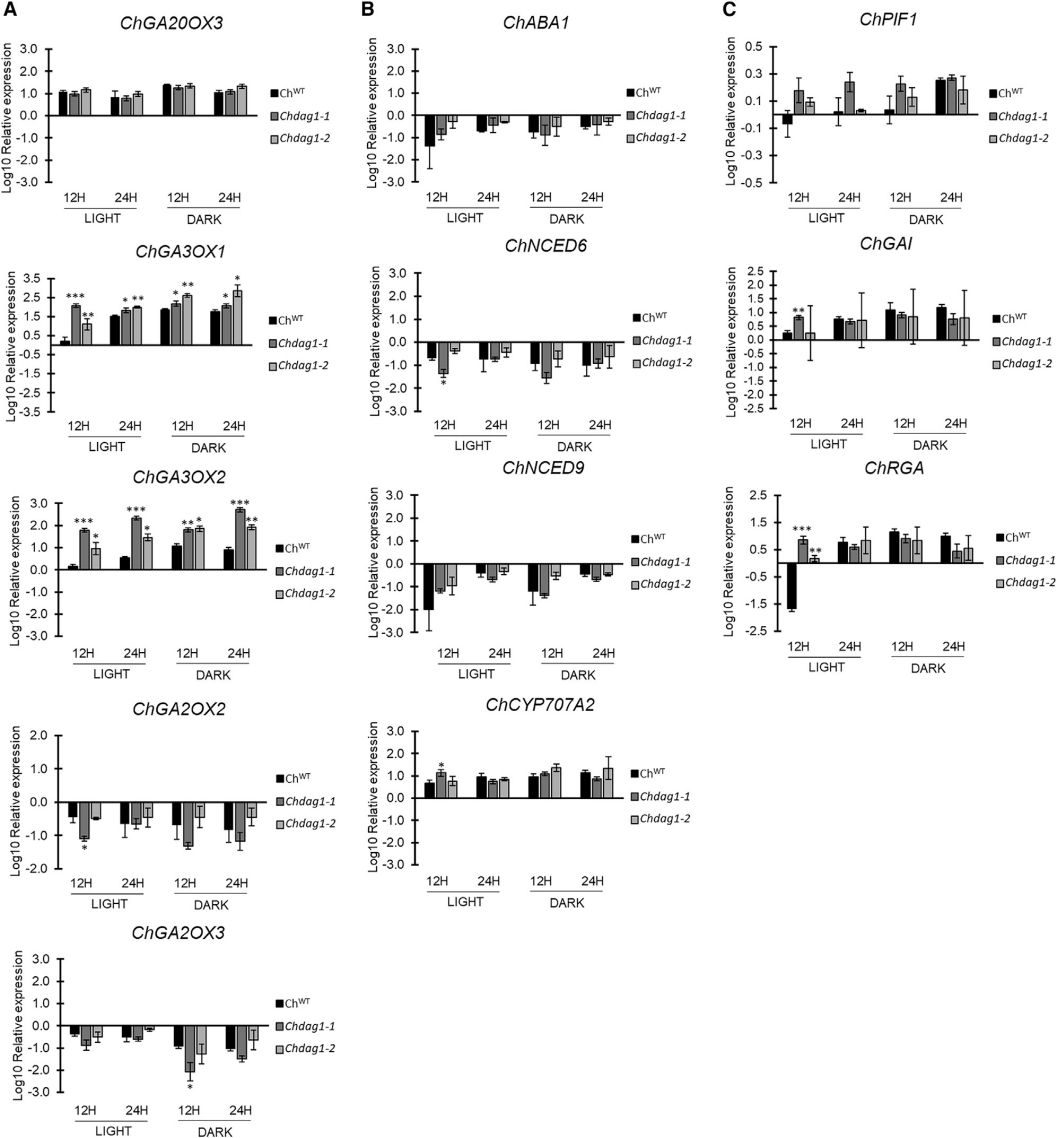
Expression profiles of GA and ABA genes in *Chdag1-1* and *Chdag1-2* mutant seeds. **(A–C)** Relative expression level of: *ChGA20OX3*, *ChGA3OX1*, *ChGA3OX2*, *ChGA2OX2*, *ChGA2OX3***(A)**, *ChABA1*, *ChNCED6*, *ChNCED9*, *ChCYP707A2***(B)**, *ChPIF1*, *ChGAI*, *ChRGA***(C)**, in *Chdag1-1* and *Chdag1-2* mutant seeds compared to the wild-type, at 12 and 24 HAI (Hours After Imbibition), under light and dark conditions. Expression levels as log10 respect to the dry condition, set to 0 (X axis). The values of relative expression levels are means of three biological replicates, with SD values. Significant differences were analyzed by *t*-test (∗∗*p* ≤ 0.001, ∗∗*p* ≤ 0.005, ∗*p* ≤ 0.05).

Given that ChDAG1 is a transcription factor and its homolog AtDAG1 functions as repressor of *AtGA3OX1* and *CYP707A2* (Gabriele et al., 2010; Boccaccini et al., 2016), we wondered whether inactivation of *ChDAG1* could affect the expression of GA and ABA metabolic genes. Therefore, the transcript levels of *ChGA20OX3*, *ChGA3OX1*, *ChGA3OX2*, *ChGA2OX2*, and *ChGA2OX3* for GA, and of *ChABA1*, *ChNCED6*, *ChNCED9*, and *ChCYP707A2* for ABA were measured in dry conditions, and in 12- or 24-h imbibed wild-type and both *Chdag1-1* and *Chdag1-2* mutant seeds, either exposed to white light or kept in total darkness. The results of this analysis showed that lack of DAG1 results in the upregulation of the GA biosynthetic genes *ChGA3OX1* and *ChGA3OX2* (Figure 4A). In particular, in *Chdag1-1* seeds *ChGA3OX1* transcript level was extremely higher at 12 HAI in light (23-fold, compared to the wild-type) and increased 2-fold also at 24 HAI, and at both 12 and 24 HAI in the dark, although to a lesser extent (2.3- and 2-fold, respectively). Surprisingly, the transcript level of *ChGA3OX2* showed an even more relevant upregulation compared to the wild-type, with a 50- and 63-fold increase at 12 and 24 HAI in the light, and a 6- and 61-fold increase under dark conditions at 12 and 24 HAI, respectively (Figure 4A). These results were corroborated by the expression profile of these genes in the *Chdag1-2* allele, which perfectly matched the one of *Chdag1-1.* Indeed, *ChGA3OX1* transcript level increased 3-fold at 12 and 24 HAI in the light, and 6- and 12-fold in dark conditions, while expression of *ChGA3OX2* increased 8- and 9-fold in the light, and 7- and 11-fold in the dark (12 and 24 HAI, respectively) (Figure 4A).

**Figure 5:**
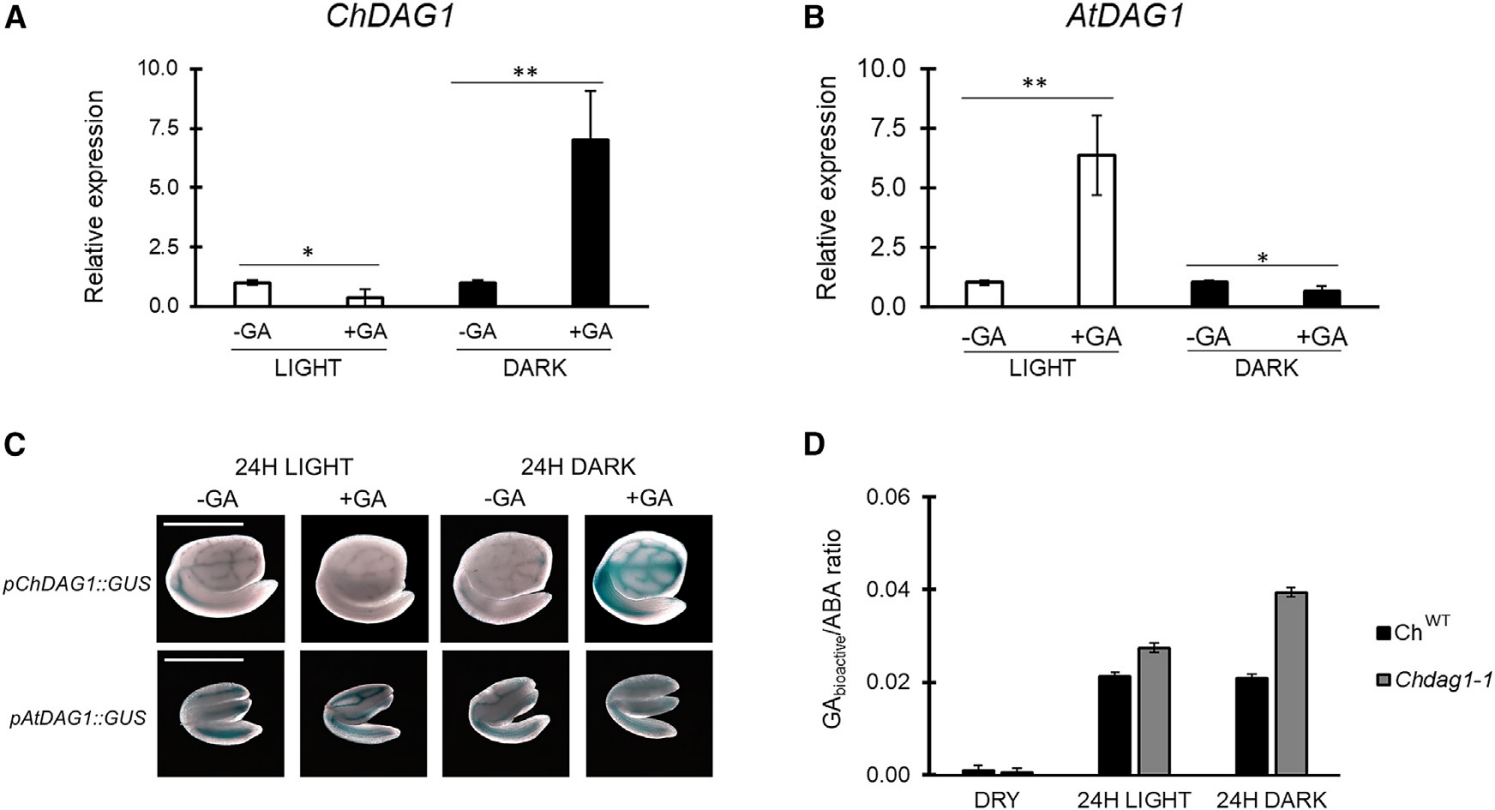
GAs promote *ChDAG1* expression in dark-imbibed seeds. **(A and B)** Relative expression level of *ChDAG1***(A)** and *AtDAG1***(B)** in 24-h imbibed wild-type seeds (Ox and Ws, respectively), in the presence of water (control) or GA_4+7_ (100 μM), in white light or in darkness. The values of relative expression levels are the mean of three biological replicates, with SD values. Expression levels were normalized with that of the *ChUBQ10* and *AtUBQ10* genes for *Cardamine* and *Arabidopsis* samples, respectively. The values are the mean of three biological replicates, with SD values. Significant differences were analyzed by *t*-test (∗∗*p* ≤ 0.005, ∗*p* ≤ 0.05). **(C)** Histochemical staining of *pChDAG1::GUS* and of *pAtDAG1::GUS* seeds imbibed 24 h, with/without addition of GAs, under white light (WL) or in dark (D). Scale bar, 1 mm. **(D)** Ratio of bioactive GAs/ABA in *Chdag1-1* mutant seeds compared to Ox seeds (Figure 2D). The analyses were performed on dry and 24-h imbibed seeds in light and dark conditions. The values are the mean of three biological replicates, with SD values.

On the other hand, expression of ABA metabolic genes was not affected by the inactivation of *ChDAG1*, as the transcript level of *ChABA1*, *ChNCED6*, *ChNCED9*, and *ChCYP707A2* was comparable in *Chdag1* mutant alleles and wild-type seeds (Figure 4B and supplemental Figure 9B), thus suggesting that ChDAG1 activity is specifically aimed at controlling GA levels. To test this hypothesis, we measured the GA levels in *Chdag1-1* seeds compared to wild-type seeds. This experiment revealed a complex fine-tuning of GA levels, with lack of ChDAG1 resulting in increased levels of bioactive GA_5_ and GA_6_, unlike GA_1_ or GA_4_ (supplemental Figure 8). Nevertheless, the GA/ABA ratio was increased in *Chdag1-1*-imbibed seeds in dark and light conditions, compared to wild-type seeds (Figure 5D). These data indicate that a differential fine-tuned regulation of the levels of GAs by ChDAG1 contributes to the different germination activity of *Arabidopsis* and *Cardamine* seeds. This hypothesis is also supported by the expression profile of *ChGAI* and *ChRGA* in the *Chdag1* mutant background, as these *DELLA* genes are upregulated in light-imbibed seeds at 12 HAI, suggesting that ChDAG1 is required to repress their expression. Intriguingly, the inactivation of *ChDAG1* results in higher steady-state level of *ChGAI* and *ChRGA* at 12 HAI in the light, making the expression in light- and dark-imbibed seeds not significantly different as it is in wild-type seeds (Figure 4C).

### *ChDAG1* expression is induced by GAs in the dark

As a fine-tuning of GA metabolism is fundamental for germination in dark conditions and ChDAG1 is able to control GA homeostasis, we questioned whether GAs might influence ChDAG1 levels to permit light-independent germination.

Therefore, we used real-time qPCR to measure *ChDAG1* transcript level in seeds imbibed in the presence of GAs, in white light or in total darkness. Interestingly, expression of *ChDAG1* was significantly increased by GAs in seeds kept in the dark (up to almost 8-fold compared to the control set to 1), while it was downregulated by GAs in light-imbibed seeds (up to 5.3-fold) (Figure 5A). Conversely, *AtDAG1* was induced to the same extent (8-fold) in *Arabidopsis* wild-type seeds imbibed in the presence of GAs in white light, as expected (Boccaccini et al., 2016), while *AtDAG1* transcript level decreased (almost 2-fold) when seeds were imbibed with GAs in the dark (Figure 5B). To further scrutinize this result, we exploited *pChDAG1::GUS Cardamine* and *pAtDAG1::GUS* transgenic lines and we analyzed the whole-mount expression of *ChDAG1* and *AtDAG1* on seeds imbibed 24 h in the presence/absence of GAs (GA_4+7_ 100 μM), in light or dark conditions. Consistently, GUS staining was notably expanded in all the vascular tissue in late embryos of *pChDAG1::GUS* seeds imbibed in the presence of GAs in the dark, compared to seeds with GAs in white light, or with those without GAs regardless of light conditions, whereas *pAtDAG1::GUS* showed increased GUS staining in light-imbibed seeds (Figure 5C). Our data suggest that GA enhances *ChDAG1* expression. Since ChDAG1 is a potential repressor of *ChGA3OX1* and *2*, while its expression is induced by GAs, a feedback control of GA levels can be hypothesized.

### To what extent are ChDAG1 and AtDAG1 functional homologs?

We then investigated whether and how ChDAG1 might act in a different fashion from the AtDAG1.

**Figure 6:**
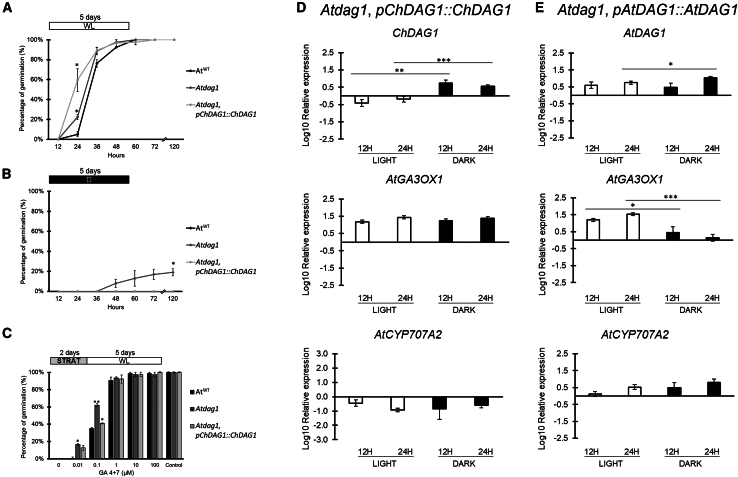
Activity of ChDAG1 in the *Atdag1* background. **(A–C)** Germination rates of *Atdag1*,*pChDAG1::ChDAG1*, *Atdag1*, and wild-type (Ws) seeds in white light **(A)**, in total darkness **(B)**, and in the presence of PAC 100 μM + increasing concentrations of GA_4+7_**(C)**. Germination rates were measured at different HAI (12, 24, 36, 48, 60, 72, and 120) in **(A** **and B)**, and at 120 HAI in **(C)**. The values are the mean of three biological replicates, with SD values. Significant differences were analyzed by *t*-test (∗∗*p* ≤ 0.005, ∗*p* ≤ 0.05). PAC, paclobutrazol; HAI, hours after imbibition. Control is referred to “mock treatment control” with ethanol. The diagram on top depicts the light treatment scheme; STRAT, stratification (2 days at 4°C, dark), WL, white light; D, dark. **(D and E)** Relative expression level of *ChDAG1* and *AtDAG1***(D** **and E)** and of *AtGA3OX1* and *AtCYP707A2* (from top to bottom). Seeds of *Atdag1*,*pChDAG1::ChDAG1-a***(D)** and *Atdag1*,*pAtDAG1::AtDAG1-a***(E)** at 12 and 24 HAI, under light and dark conditions. Expression levels as log10 respect to the dry condition, set to 0 (X axis). The values of relative expression levels are means of three biological replicates, with SD values. Significant differences were analyzed by *t*-test (∗∗∗*p* ≤ 0.001, ∗∗*p* ≤ 0.005, ∗*p* ≤ 0.05).

Indeed, the *Cardamine* DAG1 protein shares 91.4% amino acid identity with AtDAG1, suggesting that the two proteins may be functionally homologous, at least to a certain extent. Therefore, to evaluate this potential functional homology, we produced the transgenic lines expressing on one hand *ChDAG1* in the *Arabidopsis dag1* mutant, and on the other hand the fully complementing *AtDAG1* in the same background as control. The two constructs contained the *ChDAG1* and *AtDAG1* genomic loci under the control of 2.15 kb of their own promoters. Four transgenic lines for each construct have been selected and analyzed; the results of two lines for each construct are shown (Figure 6 and supplemental Figure 9) The phenotypic analysis for seed germination of the *Atdag1* mutant expressing *ChDAG1* (named *Atdag1*,*pChDAG1::ChDAG1*) revealed that ChDAG1 was unable to complement the phenotype of *Atdag1* in the light. Indeed, as expected (Papi et al., 2000), *Atdag1* mutant seeds germinated faster than *Arabidopsis* wild-type seeds (23% vs. 5% at 24 HAI), while mutant seeds expressing *ChDAG1* showed an even increased germination rate at 24 HAI (60%) (Figures 3E and 6A and supplemental Figure 9A). Conversely, expression of *ChDAG1* in the *Atdag1* background was able to complement the dark germination mutant phenotype (Papi et al., 2000), since germination dropped to 0, as in wild-type seeds (Figure 6B and supplemental Figure 9B). With respect to GA requirement, *Atdag1* mutant seeds showed increased germination frequencies compared to wild-type seeds (17% vs. 1% and 62% vs. 35% at 0.01 and 0.1 μM GA, respectively), consistently with previous results (Gualberti et al., 2002). Similarly, *Atdag1* expressing *ChDAG1* showed a similar germination trend, although with a slightly reduced germination rate at 0.1 μM GA (41%), indicating that again ChDAG1 was unable to revert the *Atdag1* phenotype (Figure 6C and supplemental Figure 9C).

We analyzed the expression of *ChDAG1* and found that in the *Atdag1* mutant background was lower during imbibition in the light with respect to dry seeds, while it was higher in the dark (Figure 6D and supplemental Figure 9D), suggesting a different transcriptional control of *ChDAG1* compared to *AtDAG1*. This would explain the inability of ChDAG1 to complement the white light phenotypes of the *Atdag1* mutant. To verify whether ChDAG1 can complement the function of AtDAG1 in repressing *AtGA3OX1* and *AtCYP707A2*, we measured the expression level of these genes in *Atdag1*,*pChDAG1::ChDAG1* and in the *Atdag1*,*pAtDAG1::AtDAG1* complemented line, as a control. In addition, the expression of *AtGA3OX2*, *AtGA2OX2*, *AtGA2OX3*, *AtABA1*, *AtNCED6* and *AtNCED9* was also evaluated (supplemental Figure 10). Surprisingly, in the dark, *AtGA3OX1* was upregulated in the *Atdag1*,*pChDAG1::ChDAG1* lines while, as expected, it was repressed in the *Atdag1*,*pAtDAG1::AtDAG1* complemented line. This indicates that, despite the high amino acid identity between AtDAG1 and ChDAG1, the latter is unable to repress *AtGA3OX1* (Figure 6E, top, and supplemental Figure 9E, top). Expression of *AtCYP707A2* was downregulated by ChDAG1 in *Atdag1*,*pChDAG1::ChDAG1* seeds, both in light and dark conditions, compared to the complemented line, suggesting an over-repression by ChDAG1 on this ABA catabolic gene (Figure 6E, bottom, and supplemental Figure 9E, bottom). On the other hand, expression of *ChDAG1* in the *Atdag1* background did not affect expression of any other GA and ABA metabolic genes, as the expression profiles in *Atdag1*,*pChDAG1::ChDAG1* were similar to the ones in the *Atdag1*,*pAtDAG1::AtDAG1* complemented line (supplemental Figure 10).

Our results support the idea that AtDAG1 and ChDAG1, despite having partial overlapping functions, might control homeostasis of GAs in a different fashion. Moreover, our results highlight the possibility that, despite orthologs, the regulation of the expression of *ChDAG1* and *AtDAG1* might differ in the two species.

## Discussion

Seed germination represents a critical process in the life cycle of a plant, and in particular for plant adaptation to changing environmental conditions. Flowering plants have evolved several adaptive traits to ensure successful germination, such as the need for light, especially for small-seed species, which tend to prefer their dispersed seeds to be not too deep into the soil (Pons, 2000). Utilizing two close relatives’ model systems with different light dependency for breaking seed dormancy, *Arabidopsis* and *Cardamine*, we uncovered the key and conserved molecular mechanisms allowing germination in the dark after imbibition. In particular, we established a fundamental role for GA metabolism regulation by the repressor ChDAG1 in dark conditions that enables light-independent germination.

### Dark germination is dependent on a high GA/ABA ratio

The role of the photoreceptor phyB is well-established in triggering GA biosynthesis (Derkx and Karssen, 1994; Toyomasu et al., 1998; Yamaguchi et al., 1998; Yamaguchi and Kamiya, 2000; García-Martinez and Gil, 2001; Koornneef et al., 2002; Ogawa et al., 2003) as well as in increasing GA sensitivity (Hilhorst and Karssen, 1988; Yang et al., 1995) during seed germination. On the other hand, phytochromes negatively control ABA levels in seeds, as R light treatment decreases the transcript level of ABA biosynthetic genes while increasing expression of the *CYP707A*-encoding genes (Seo et al., 2006; Oh et al., 2007; Sawada et al., 2008).

Our data provide compelling evidence that dark germination in *Cardamine* is enabled by the maintenance of proper GA content in dark-imbibed seeds, which results from highly expressed GA biosynthetic genes and downregulated catabolic ones. These results are quite consistent with those obtained by Mérai et al. (2019), in two accessions of *Aethionema arabicum*. Indeed, they showed that *GA3OX1* and *2* were more expressed in the dark, both in the accession with dark-dependent germination (Cyprus) and in the one with light-neutral germination (Turkey). In contrast, the catabolic *GA2OX3* gene was downregulated (Mérai et al., 2019), thus strengthening the notion that GA levels are crucial for germination in the absence of light. Consistently, overexpression of *GA2OX2* in *Cardamine* seeds results in a significant decrease of the germination percentage. Interestingly, inactivation of *AtGA2OX2*, the only gibberellin 2-oxidase-encoding gene expressed in *Arabidopsis* seeds in the dark (Ogawa et al., 2003), resulted in increased germination rate in the dark (Yamauchi et al., 2007). This evidence enables to posit that fine-tuning GA levels might be a conserved mechanism, which would then have evolved in plants with light-dependent germination. Corroborating this hypothesis, GA signaling pathways and their molecular mechanism are conserved in seed plants (Hedden, 2003; Sun and Gubler, 2004). Differently several spore plants such as *Physcomitrella patens* do not present functionally conserved GA signaling elements, supporting our evolutionary hypothesis (Schwechheimer and Willige, 2009). Indeed, this bryophyte possesses a functional GA receptor, a homolog of the *Arabidopsis* GID1, and a DELLA protein, that is able to interact with the GID1-GA complex and is degraded in a GA-dependent manner (Hirano et al., 2007; Yasumura et al., 2007).

Noteworthy, a very similar expression profile for GA metabolic genes is present in *Lepidium* but not in *Capsella*, a species with light-dependent germination (this work). Indeed, although expression of the biosynthetic genes *LsGA3OX1* and *2* are higher in light- than in dark-imbibed seeds, the extremely low level of the catabolic gene should result in a high level of GAs in darkness, similarly to what occurs in *Cardamine* seeds.

Intriguingly, GA levels are unusually high in dry seeds, compared to *Arabidopsis* seeds, and consequently the amounts might seem inconsistent with the transcript levels of the biosynthetic genes at 24 HAI. In the future, it will be interesting to measure the amount of GAs and transcript levels during seed maturation.

What is fundamental to control germination is the GA/ABA ratio, which is definitely consistent with the expression data. In addition, it should be noted that, even in *Aethionema*, the data of bioactive GAs are not really consistent, since if GA4 is coherent with *GA3OX1* transcript level and with the germination rates of the two accessions, GA6 is extremely higher in dark and light CYP seeds, inconsistently with the germination rates (Mérai et al., 2019).

Also, the results concerning ABA, which counteracts the germination-promoting function of GAs, are extremely consistent with the dark germination ability of *Cardamine* seeds. Indeed, ABA biosynthetic genes are downregulated during imbibition, regardless of light conditions, in contrast to dry seeds, which show high transcript levels of *ChABA1*, *ChNCED6*, and *ChNCED9.* On the other hand, the transcript levels of *CYP707A2*, the main catabolic gene in *Cardamine* seeds as in *Arabidopsis* (Okamoto et al., 2006), are elevated during seed imbibition, similarly in the dark and in the light. In agreement with these expression data, ABA levels in dry seeds are extremely high, then at 24 HAI they decrease, both in dark and in light. Presumably, this is necessary to establish and maintain seed dormancy, which in *Cardamine* seeds is similar to *Arabidopsis*. We cannot exclude that ABA might act through the downstream ABA HYPERSENSITIVE GERMINATION 1 and 3 (AHG1/3) PP2C phosphatases (Yoshida et al., 2006; Nishimura et al., 2007), nor that the dormancy-promoting factor DELAY OF GERMINATION 1 (DOG1) may function on a pathway parallel to ABA converging on these phosphatases, as has been recently proved in *Arabidopsis* (Née et al., 2017; Nishimura et al., 2018).

In *Arabidopsis* and most annual plants, a number of negative regulators work to prevent germination in the dark, such as the master repressor PIF1, the DELLA proteins, GAI and RGA, and the Dof transcription factor DAG1 (Oh et al., 2006, 2007; Gabriele et al., 2010). Given that these genes are highly conserved among Brassicaceae, studying their function in light-independent or light-inhibited species represents a crucial step to unravel the molecular mechanisms underlying this process when not mediated by light. Comparing transcript levels in dried vs. imbibed seeds we reveal that only the steady-state level of the *DAG1* transcript is significantly higher in dry seeds than in imbibed ones. This is in contrast to *Arabidopsis*, where *AtDAG1* is induced during imbibition, thus suggesting a putative role of ChDAG1 in the control of seed germination independently of both PIF1 and light. On the other hand, *PIF1* expression was extremely low in all conditions, except at 24 HAI in the dark, which does not rule out that it may have post-translational regulation as in *Arabidopsis*, as has also been presumed in *Aethionema arabicum* (Mérai et al., 2019). Also *GAI* and *RGA* have a similar expression profile in *Cardamine* than in *Arabidopsis* seeds, which is consistent with their role as repressor of the GA-mediated germination process (Oh et al., 2007; Davière and Achard, 2013).

### DAG1 as the factor tuning down GA levels

In *Arabidopsis* seeds, DAG1 fine-tunes the GA/ABA balance irrespective of light conditions, but also the GA homeostasis, through a feedback loop on *AtGA3OX1* (Boccaccini et al., 2016). Of 29 *Cardamine* putative *DOF* genes, only the protein encoded by the single-copy gene *ChDAG1* shares 94,1% amino acid identity with AtDAG1, thus we hypothesized that ChDAG1 might be involved in the control of light-independent seed germination in *Cardamine*. Indeed, the inactivation of *ChDAG1* affects seed germination: *Chdag1* seeds display a faster germination kinetics, similarly to *Atdag1* seeds (Papi et al., 2000), while having an increased GA requirement, in contrast to *Atdag1* (Gualberti et al., 2002). As for ABA, ChDAG1, unlike AtDAG1, is not likely to function on ABA levels, as revealed by the similar expression of ABA metabolic genes as well as by the comparable amount of ABA in *Chdag1-1* and wild-type seeds. Thus, ChDAG1 seems to be committed only to the repression of the two key GA biosynthetic genes which, in the absence of ChDAG1, are upregulated, particularly in light-imbibed seeds. Interestingly, *AtGA3OX1* is a direct target of AtDAG1, which represses its expression to prevent dark germination in *Arabidopsis* seeds (Gabriele et al., 2010; Boccaccini et al., 2014). This would suggest a conserved function between the two Brassicaceae species; however, surprisingly, ChDAG1 is unable to repress *AtGA3OX1*, when expressed in the *Atdag1* mutant background, as AtDAG1 does. The Dof domains of ChDAG1 and AtDAG1 are identical, indicating that ChDAG1 should recognize the Dof binding sequence, which is present on the *AtGA3OX1* promoter and bound by AtDAG1 (Gabriele et al., 2010; Boccaccini et al., 2014). However, given that AtDAG1 negatively regulates *AtGA3OX1* by cooperating with AtGAI (Boccaccini et al., 2014), one possibility is that ChDAG1 is not able to interact with AtGAI and, in turn, to bind Dof binding sites on the *AtGA3OX1* promoter to repress its expression.

Remarkably, expression of *ChDAG1* in the *Atdag1* background results in a striking and unexpected downregulation of *AtCYP707A2*, in imbibed seeds relative to dry seeds (Boccaccini et al., 2016), different from the *Arabidopsis* complemented line (*Atdag1*,*pAtDAG1::AtDAG1*). Therefore, ChDAG1 is likely to induce an over-repression of *AtCYP707A2*, as it would be expected to bind *CYP707A2* promoter constitutively. It is well established that Dof proteins can interact with other regulatory factors and these interactions contribute to the specificity of DOF proteins (Yanagisawa, 2001). We cannot rule out the hypothesis that ChDAG1 is unable to interact with a corepressor or that, rather it has an increased affinity for the *AtCYP707A2* promoter.

It should be noted that, in the *Atdag1* background, the expression profile of *ChDAG1* is different from the one of *AtDAG1* itself, indicating that: (1) the promoters of *ChDAG1* and *AtDAG1* do not share the same regulatory regions, (2) their expression is controlled by the same external/internal cues but in a different way, and (3) other levels of regulation, namely epigenetic, could take place differentially on *ChDAG1* respect to *AtDAG1*.

The *AtDAG1* and *ChDAG1* promoters share two relevant regulatory domains, an E-box and a MADS-box, which, although both associated with seed dormancy and germination (Oh et al., 2009; Yu et al., 2017), have not been linked to AtDAG1 by any evidence so far. Both *DAG1* loci are regulated by GAs, but in opposite ways; *AtDAG1* is positively controlled in the light by GAs (this work; Boccaccini et al., 2016), while in *Cardamine* seeds GAs control the expression of *ChDAG1* positively in the dark and negatively in the light. These data highlight the hypothesis that GAs act as an internal signal, both in *Arabidopsis* and *Cardamine* seeds, but with opposite effects. Therefore, the *ChDAG1* locus, once in the presence of an increased amount of GAs, as in the *Atdag1* background (Boccaccini et al., 2016), will be repressed, as it is in the light-imbibed seeds of *Atdag1*,*pChDAG1::ChDAG1.* The extremely low expression levels of *ChDAG1* in light-imbibed *Atdag1* seeds are, at least partly, the reason why ChDAG1 is unable to complement any germination phenotypes of *Atdag1.* Last, the epigenetic control, mediated by PRC2, which has been demonstrated on the *AtDAG1* locus, should also be considered (Boccaccini et al., 2016). So far there is no data on epigenetic control during *Cardamine* development. Further studies are needed to explore it even during the seed-to-seedling transition, possibly also by using the pharmacological approach that has already proven to be efficient in *Arabidopsis* (Ruta et al., 2019).

### A model of the light-independent germination process

Taken together, our physiological and molecular data provide a solid framework for dark germination in *Cardamine* seeds and, possibly, in other Brassicaceae with light-independent germination. The phenotypic and molecular characterization of ChDAG1, both in its natural context, *Cardamine* seeds, and in the context of *Arabidopsis* seeds, as a light-dependent germination species, allowed us to outline a scheme which, although still representing a working model, represent a first important step in unveiling the mechanisms underlying light-independent and, possibly, light-inhibited germination.

Although many plant species, including *Arabidopsis*, can germinate in total darkness, the ecological context of their germination differ significantly from *Cardamine hirsuta*. Indeed, wild-type *Arabidopsis* seeds mainly depend on light-activated phytochrome pathways for germination, and, consistently, it was necessary to screen over 300 *Arabidopsis* accessions to identify 3 QTLs required for increasing germination under cold and dark (Meng et al., 2008). Similarly to *Cardamine hirsuta*, *Aethionema arabicum* can germinate in darkness, but its utility as a model system is limited by the evolutionary distance from *Arabidopsis thaliana* and lack of genetic tractability. Comparative genomic studies suggest that *Cardamine hirsuta* shares approximately 85%–90% genomic similarity with *A. thaliana* (Gan et al., 2016), while *Aethionema arabicum* shares 70%–80% genomic similarity (Mérai et al., 2019). This restricts relevance of this species for direct comparative studies and functional genomic analyses. In contrast, *C. hirsuta* is evolutionarily closer to *Arabidopsis* and amenable to genetic transformation, making it a more suitable model for studying light-independent germination.

**Figure 7:**
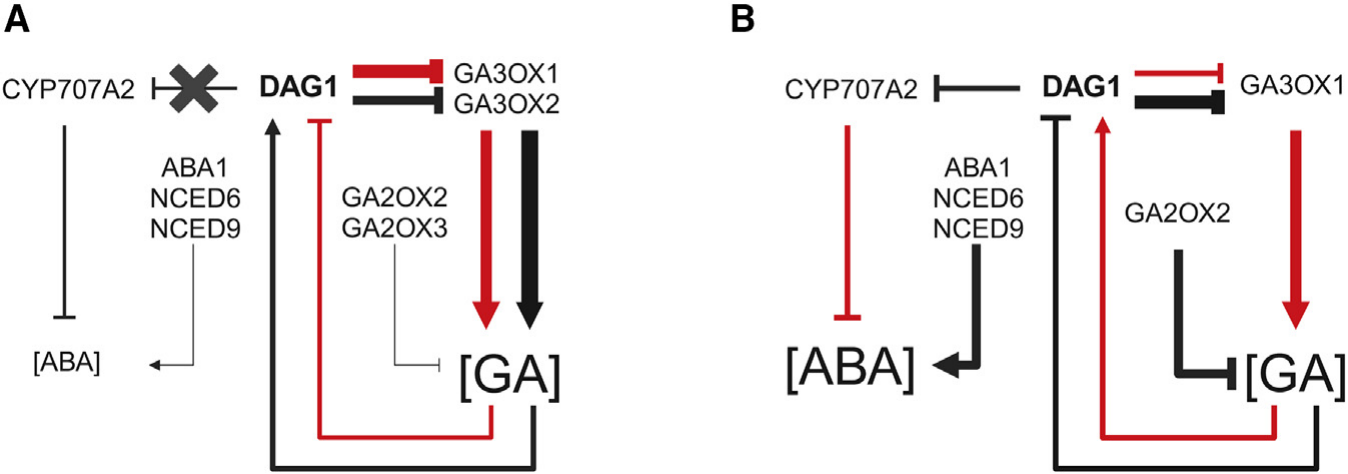
Scheme of the molecular mechanism underlying seed germination in *Cardamine*. **(A and B)** Scheme of the main elements involved in seed germination in *Cardamine***(A)** and *Arabidopsis***(B)**. Red and black arrows are referred to light and dark conditions, respectively. The arrows' thickness is referred to the expression level of the corresponding genes. **(A)** In seeds of *Cardamine*, the transcript levels of *ChGA3OX1* and *ChGA3OX2* are higher in the dark than in the light, thus increasing GA levels. ChDAG1 represses these two GAs’ biosynthetic genes which, in the absence of ChDAG1, are upregulated, particularly in light-imbibed seeds. The transcript level of *ChCYP707A2* is not altered by the inactivation of *ChDAG1*, suggesting that ChDAG1 is not involved in its regulation. GAs increase *ChDAG1* transcript level in the dark, while decreasing it in the light. **(B)** In seeds of *Arabidopsis*, AtDAG1 represses both *AtGA3OX1* and *AtCYP707A2*, mainly in the dark (Gabriele et al., 2010; Boccaccini et al., 2016). GAs increase *AtDAG1* transcript level in the light, while decreasing it in the dark.

In this model, the amount of GAs in the dark plays a key role, and, in turn, the GA/ABA ratio which is higher in dark- than in light-imbibed seeds, eliciting seed germination (Figure 7A). In this context, the importance of the role of ChDAG1 is highlighted by the control of its expression by GAs; indeed, GAs increase *ChDAG1* transcript level in the dark, while decreasing it in the light, a kind of control that mirrors what occurs in *Arabidopsis* seeds (Figure 7B).

A crucial element of this scheme is related to the DELLA proteins GAI and RGA, and to their function with respect to GAs, and to their possible interaction with DAG1. Indeed, in *Cardamine* seeds in the dark, *GAI* and *RGA* are more expressed than in the light, similarly to *Arabidopsis*, but, unlike *Arabidopsis*, GA levels are as high as in the light. Remarkably, the inactivation of *ChDAG1* results in an increase of *GAI* and *RGA* transcript levels in the light but a decrease in the dark, thus suggesting that ChDAG1 has a prominent role in the control of GA homeostasis, also fine-tuning GAI and RGA levels. On the other hand, in *Arabidopsis* seeds, AtDAG1 and GAI mutually regulate their expression to cooperate in the repression of the GA biosynthetic gene *AtGA3OX1* (Boccaccini et al., 2014).

Our data do not resolve the evolutionary and ecological reasons for the differences in light dependency germination among plants. *Cardamine* is a broadly diffused species with higher potential for adaptation to diverse environments than *Arabidopsis* (Hay et al., 2014; Baumgarten et al., 2023). *Cardamine* light-independent germination might have contributed to this adaptative potential, permitting to colonize the most diverse environments. Future studies on seed germination of different *Cardamine* genotypes might help to uncover the evolutive history of this trait and how this contributed to colonization success. From a physiological point of view, eventual differences in the composition of the cell wall might have evolved causing differences in the mechanical and chemical properties that are fundamental during seed germination in both light and dark conditions and are linked to GA levels (Chen and Bradford, 2000; Sinclair et al., 2017; Xu et al., 2020). Considering this hypothesis, further studies utilizing *Cardamine* as a model system are required to provide evidence on this fundamental topic.

In conclusion, our data represent a step forward in our understanding of dark germination, a topic of high interest in biotechnology including in all the conditions where light accessibility is limited, as in the fascinating story of space exploration.

## Methods

### Plant material and growth conditions

All the seeds from *Cardamine hirsuta* (ecotype Oxford), *Arabidopsis thaliana* (ecotype Wassilewskija or Columbia), *Capsella rubella*, *Lepidium sativum*, and *Camelina sativa* used in this work were grown in a growth chamber at 24°C/21°C with 16/8-h day/night cycles and light intensity of 300 μmol/m^−2^ s^−1^ (CCT 5700 K) as described previously (Papi et al., 2000). The *Atdag1* mutant (Ws) is described by Papi et al. (2000), *phyB-9* and *phyA-201* mutant alleles are described by Reed et al. (1993, 1994).

### Seed germination assays

Seeds were harvested from completely dried mature plants grown at the same time, in the same conditions and stored for at least 4 weeks. Three different seed stocks were used for the germination assays. Twenty seeds for each genotype were sown on filter papers 595 (Schleicher & Schüll, Dassel, Germany), soaked with 5 ml water, under dim-green safe light. As for ABA and PAC assays, seeds were sown on medium containing ABA (Duchefa A0941) or PAC (Duchefa P0922), stratified, then transferred in the growth chamber and checked after 120 h. Seeds and seedlings pictures were taken with a Leica MZ12 stereomicroscope using an AxioCam ERc5s camera.

For light-pulse experiments, stratified seeds were exposed to a pulse of red light (λ = 660 nM) (40 μmol/m^−2^ s^−1^), or far-red light (λ = 735 nM) (10 μmol/m^−2^s^−1^) (mounting Heliospectra LX60 lamp), or to R-FR or R-FR-R 5′ pulses, then grown in either continuous monochromatic white light (300 μmol m^−2^ s^−1^) (CCT 5700 K) or in the dark for 120 h. The images of the video of *Arabidopsis* and *Cardamine* germinating seeds were acquired at 1 h intervals, with a custom IR imaging setup.

### Expression analysis

RNA was isolated according to Penfield et al. (2005) and Gabriele et al. (2010). RNA was purified according to the manufacturer’s protocol (NORGEN 17200). Total RNA was reverse transcribed using the PrimeScript RT reagent kit with gDNA Eraser (TaKaRa, San Jose, CA). Real-time qPCR was performed with SYBR green I master using the Rotor-Gene Q instrument (QIAGEN, Hilden, Germany). A total of 1 μl of the diluted cDNA was used, along with the specific primers (Table S1). Relative expression levels were normalized with the *UBQ10* reference gene and, unless otherwise stated, were presented as log10 of relative expression compared to dry seeds, which was set to 0 (indicated by the X axis).

### Generation of transgenic plants

The CRISPR-Cas9 mutant was obtained following the protocol of Schiml et al. (2016). A single-guide RNA (sgRNA) was designed using the CRISPR-Cas9 target online predictor “CCTop” (https://cctop.cos.uni-heidelberg.de/), and the sgRNA with low chance of causing off-target effects was selected. The sgRNA was assembled in a pEn-Chimera vector, then transferred by Gateway reaction to pmr284 vector, coding the Cas9 protein. *Cardamine hirsuta* Ox plants were transformed by *Agrobacterium* using the floral dip method. Sequences targeted by sgRNA of T1 mutant plants were examined by TIDE (Tracking of Indels by Decomposition) analysis. T3 progeny of Cas9-free homozygous plants were selected for both the *Chdag1-1* and *Chdag1-2* alleles.

For cloning of the constructs *pAtDAG1::AtDAG1* and *pChDAG1::ChDAG1*, the Gateway system (Invitrogen) was used. The genomic sequences of *AtDAG1* and *ChDAG1* and the 2 kb region upstream were amplified from *Arabidopsis thaliana* (Ws) and *Cardamine hirsuta* (Ox), respectively, using the primers listed in Table S1.

To generate *ChUB10::GAL4* construct, pDONORP4P1-pChUB10 (Di Ruocco et al., 2018) and pDONOR221-GAL4 were recombined with pDONOR P2P3-NOS into a pB7m34GW destination vector via LR reaction (Invitrogen). The *UBQ10::GAL4*,*UAS::GA2ox2* line for the transactivation assays was obtained by crossing the single homozygous lines.

The constructs were introduced in *A. tumefaciens*, GV301. Both *Arabidopsis* and *Cardamine* plants were transformed by floral dipping (Clough and Bent, 1998; Zhang et al., 2006). For each construct, several transformants have been selected and analyzed.

### GUS construct and analysis

A 2.15-kb fragment of the *ChDAG1* promoter region amplified by PCR with *Hin*dIII and *Bam*HI restriction sites was cloned in *Hin*dIII-*Bam*HI linearized binary vector pBI101. *Cardamine* wild-type plants were transformed and several independent transformants were selected on kanamycin (50 μg/ml). Seeds were imbibed for 24 h with/without GA_4+7_ 100 μM, in dark or light conditions. GUS assay was performed according to Moubayidin et al. (2013). Samples were imaged with an Axioskop 2 plus microscope using the AxioCam ERc5s camera.

### GA and ABA determination

#### GA analysis

The analysis was performed on dry and 24-h imbibed seeds, either in light or dark conditions, for both *Cardamine* wild-type Ox and *Chdag1-1* mutant. Samples were analyzed for GA and ABA content according to Urbanová et al. (2013) with some modifications. In brief, tissue samples of about 2 mg FW were ground to a fine consistency using 2.7-mm zirconium oxide beads (Retsch, Haan, Germany) and a Precellys homogenizer (Bertin Technologies, France) with 1 ml of ice-cold 80% acetonitrile containing 5% formic acid as extraction solution. The samples were then extracted overnight at 4°C using a benchtop laboratory rotator Stuart SB3 (Bibby Scientific, Staffordshire, UK) after adding internal gibberellins standards ([^2^H_2_]GA_1_, [^2^H_2_]GA_4_, [^2^H_2_]GA_9_, [^2^H_2_]GA_19_, [^2^H_2_]GA_20_, [^2^H_2_]GA_24_, [^2^H_2_]GA_29_, [^2^H_2_]GA_34_, and [^2^H_2_]GA_44_) purchased from OlChemIm, Czech Republic. The homogenates were centrifuged at 36 670 *g* and 4°C for 10 min, and corresponding supernatants were further purified using mixed-mode SPE cartridges (Waters, Milford, MA) and analyzed by ultra-high-performance liquid chromatography-tandem mass spectrometry (UHPLC-MS/MS) (Micromass, Manchester, UK). GAs were detected using multiple-reaction monitoring mode of the transition of the ion [M–H]- to the appropriate product ion. Masslynx 4.2 software (Waters) was used to analyze the data, and the standard isotope dilution method (Rittenberg and Foster, 1940) was used to quantify the GA levels.

#### ABA analysis

Dry and 24-h imbibed seeds of *Cardamine* (wild-type Ox and *Chdag1-1* mutant) were extracted, purified, and analyzed according to a method described in Turecková et al. (2009). In brief, about 5 mg of plant tissue per sample was homogenized using a bead mill (27 Hz, 10 min, 4°C; MixerMill, Retsch, Haan, Germany) and extracted in 1 ml of ice-cold methanol/water/acetic acid (10:89:1, v/v) and internal standard (+)-3′,5′,5′,7′,7′,7′-^2^H_6_-ABA (Olchemim, Olomouc, Czech Republic). After 1 h of shaking in the dark at 4°C, the homogenates were centrifuged (36 670 *g*, 10 min, 4°C), and the pellets were then re-extracted in 0.5 ml extraction solvent for 30 min. The combined extracts were purified by solid-phase extraction on an Oasis HLB cartridges (60 mg, 3 ml, Waters), then evaporated to dryness in a Speed-Vac (UniEquip) and finally analyzed by UHPLC-ESI(–)-MS/MS. Data acquisition and analysis were performed using the MassLynx software (version 4.2, Waters).

## Funding

This work was supported by the project SEMINE - POR FESR Lazio 2014-2020 - Azione 1.2.1 - approvato con Determinazione no. G08487 del 19/07/2020 - pubblicato sul BURL N.93 del 23/07/2020 - modificato con Determinazione no. G10624/2020 - pubblicato sul BURL no. 116 del 22/09/2020 - Italy (to S.S.); by the project TowArds Next GENeration Crops, reg. no. CZ.02.01.01/00/22_008/0004581 of the ERDF Programme Johannes Amos Comenius - Czech Republic (to D.T.).

## Acknowledgments

No conflict of interest declared.

## Author contributions

R.D.I. and P.V. designed the study and supervised the study. A.L. and H.K. performed most of the experimental work with help from G.B., C.L., S.O., L.Q., M.D.V., D.S., and N.S. H.K. realized the video. D.T., V.T., and M.S. performed hormone determination. S.S. and R.D.M. analyzed the data. S.S., R.D.M., M.D.B., and P.C. discussed and commented on the study. A.L. prepared the figures. R.D.I. and P.V. wrote the paper. All authors commented on and edited the paper.

## Supplemental information

Document S1. Figures S1–S10

Table S1. Primers table

Video S1. Time-lapse video of Cardamine seeds germinating in darkness

Document S2. Article plus supplemental information
