# Supplementary material for: A DOF transcriptional repressor-gibberellin feedback loop plays a crucial role in modulating light-independent seed germination
Source: Plant Commun. 2025 Jan 28;6(4):101262. doi: 10.1016/j.xplc.2025.101262 (PMC12010397; doi:10.1016/j.xplc.2025.101262)
Supplement: Document S1. Figures S1–S10 [file mmc1.pdf]

Plant Communications, Volume *6*

Supplemental information

A DOF transcriptional repressor-gibberellin feedback loop plays a crucial role in modulating light-independent seed germination

Andrea Lepri, Hira Kazmi, Gaia Bertolotti, Chiara Longo, Sara Occhigrossi, Luca Quattrocchi, Mirko De Vivo, Daria Scintu, Noemi Svolacchia, Danuse Tarkowska, Veronika Tureckova, Miroslav Strnad, Marta Del Bianco, Riccardo Di Mambro, Paolo Costantino, Sabrina Sabatini, Raffaele Dello Ioio, and Paola Vittorioso

Plant Communications

Supplemental information

A DOF transcriptional repressor-gibberellin feedback loop is critical for modulating light-independent seed germination

Andrea Lepri, Hira Kazmi, Gaia Bertolotti, Chiara Longo, Sara Occhigrossi, Luca Quattrocchi, Mirko De Vivo, Daria Scintu, Noemi Svolacchia, Danuse Tarkowska, Veronika Tureckova, Miroslav Strnad, Marta Del Bianco, Riccardo di Mambro, Paolo Costantino, Sabrina Sabatini, Raffaele Dello Ioio, Paola Vittorioso

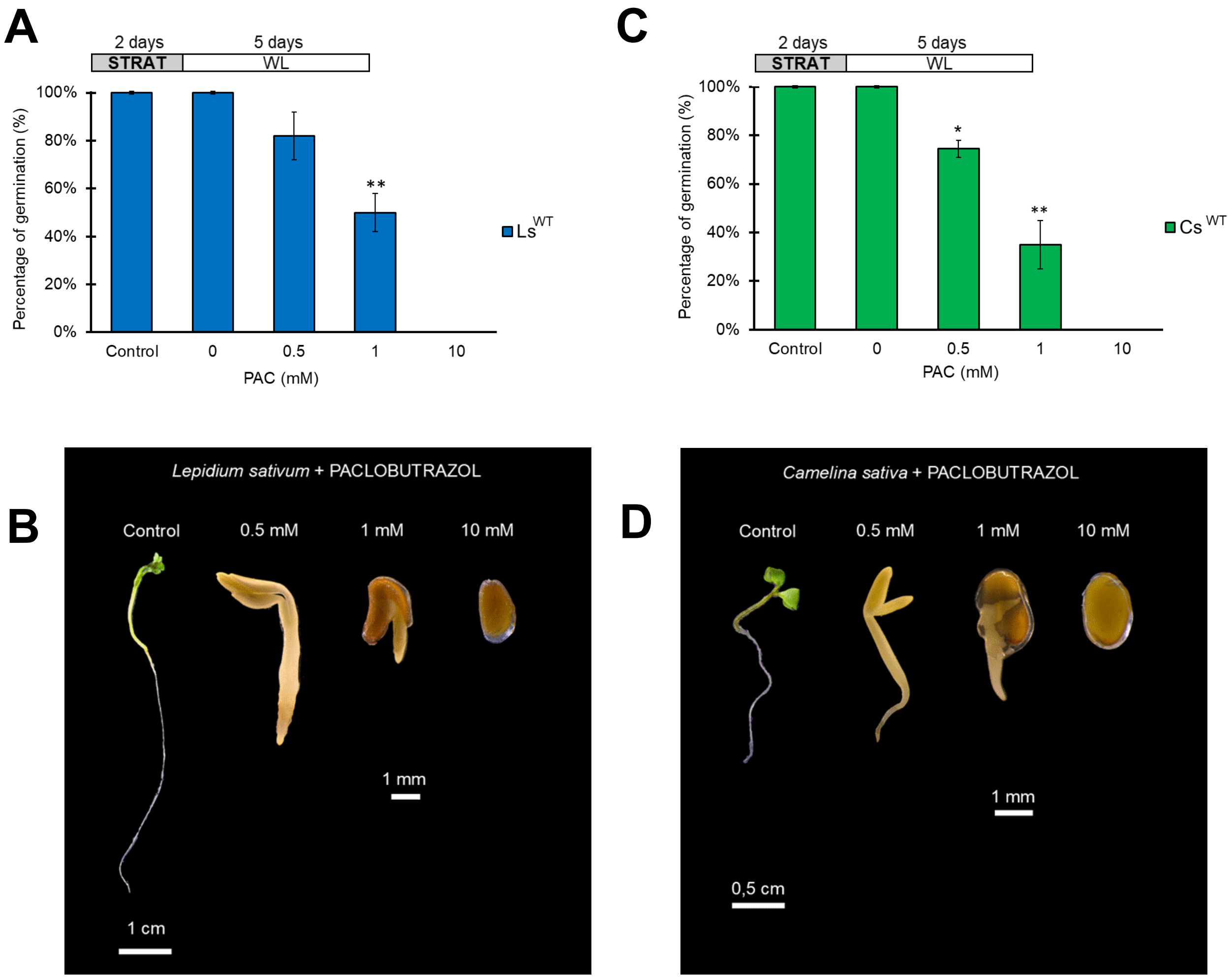

Supplemental Figure 1. PAC sensitivity of Lepidium sativum and Camelina sativa seeds.
(**A, C**) Germination rates of *Lepidium* (A) and *Camelina* (C) wild-type seeds on increasing PAC concentrations (0, 0.5, 1 and 10 mM). The values are means of three biological replicates, presented with SD values. Significant differences were analysed by one way ANOVA with post hoc Tukey multiple comparison test (**$p \leq 0.005$, * $p \leq 0.05$). Control is referred to "mock treatment control" with ethanol. The diagram on top depicts the light treatment scheme.
(**B, D**) Picture of *Lepidium* (B) and *Camelina* (D) germinated seeds on 0, 0.5, 1 and 10 mM PAC at 120 HAI. Germination rates were measured at 120 HAI. Control is referred to "mock treatment control" with ethanol. PAC: Paclobutrazol. HAI: Hours After Imbibition. The diagram on top depicts the light treatment scheme; STRAT: Stratification (2 days at 4°, Dark), WL: white light.

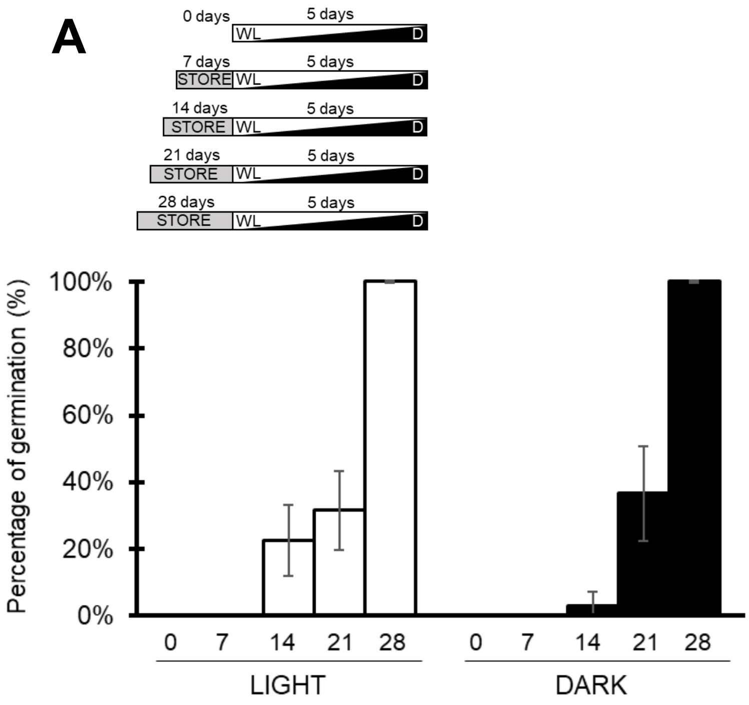

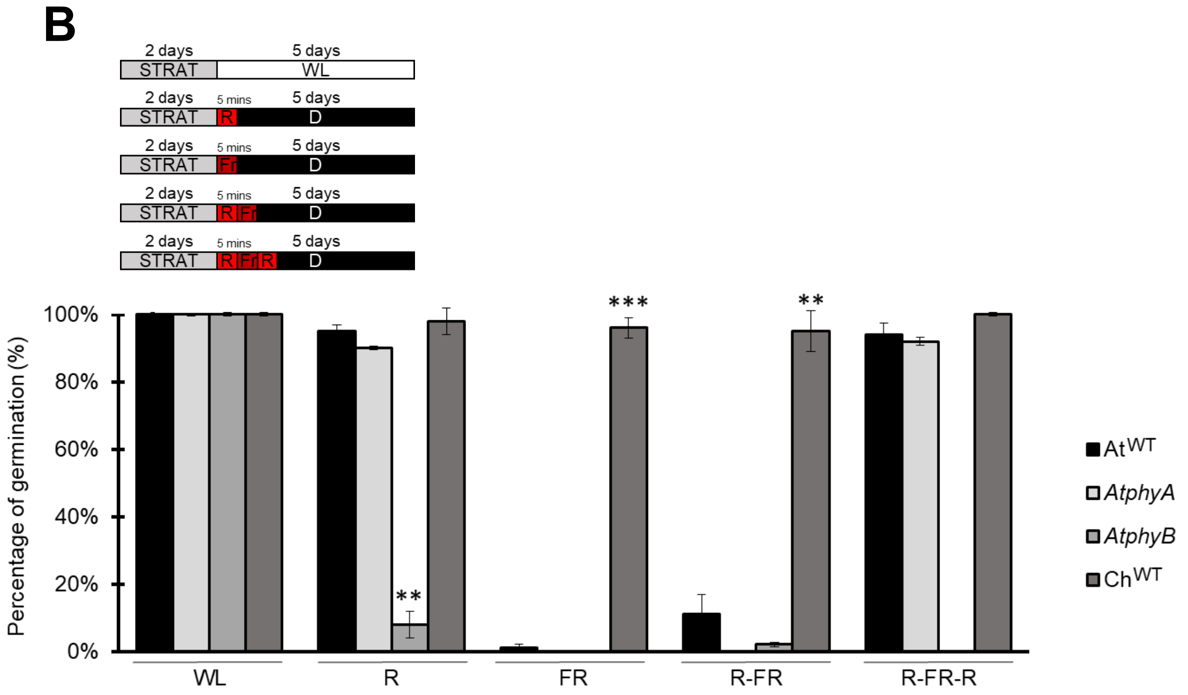

Supplemental Figure 2. Germination features of Cardamine seeds.
(**A**) Dormancy release of *Cardamine hirsuta* wild-type seeds, from freshly harvested to 4 weeks storage under light and dark conditions.
(B) Germination rates of *Cardamine hirsuta* wild-type seeds following a 5min pulse of R or Fr light, or after R-FR and R-FR-R treatments. *Arabidopsis phyA* and *phyB* mutant seeds and wild-type (Col-0) seeds were used as control of the light treatments. Germination rates were measured at 120 HAI. The values are means of three biological replicates, presented with SD values. Significant differences were analysed by t-test (****p* ≤0.001, ***p* ≤0.005). The diagram on top depicts the light treatment scheme; HAI: Hours After Imbibition, STRAT: Stratification (2 days at 4°, Dark), WL: white light, R: red light, FR: far red light, D: dark.

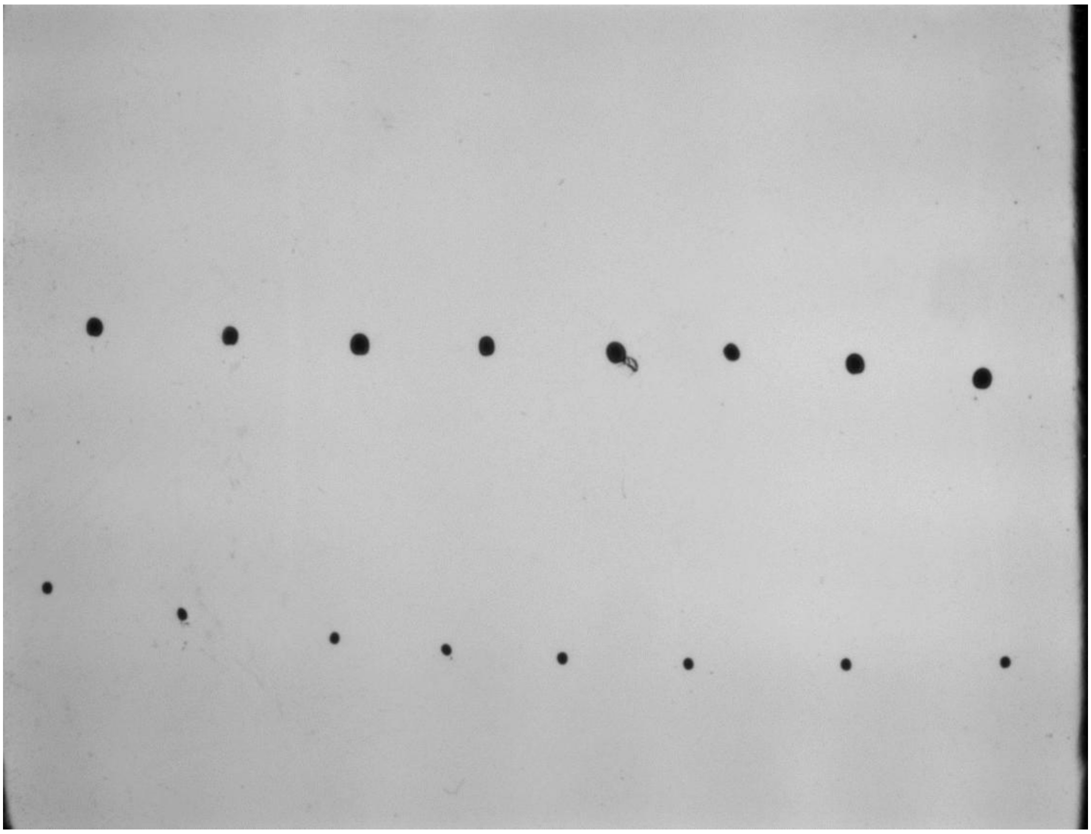

Supplemental Figure 3. Time-lapse video of Cardamine seeds germinating in darkness.
The movie has been realised with *Arabidopsis* and *Cardamine* wild-type seeds (Ws and Ox, respectively). Seeds were incubated in darkness up to 4 days.

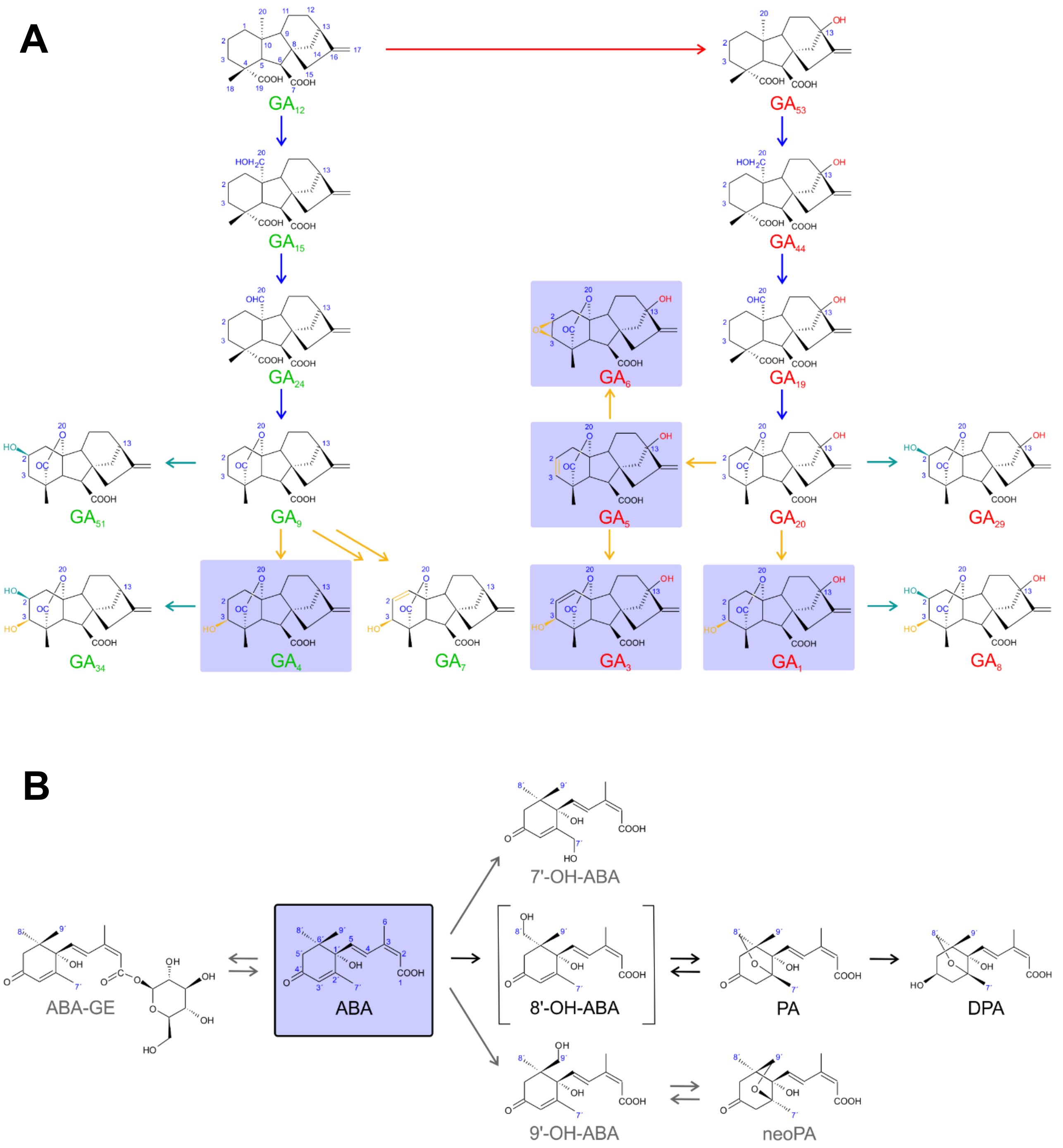

Supplemental Figure 4. GA and ABA metabolic pathways.

(A) Simplified scheme of the GAs biosynthetic and catabolic pathway. Green labelled GAs belong to the 13-nonhydroxylated pathway (leading to the production of bioactive GA4 and GA7), while red labelled GAs belong to the 13-hydroxylated pathway (leading to the formation of bioactive GA1, GA3, GA5, GA6). The bioactive GAs are indicated in coloured rectangles. The colour of the arrows indicates the type of reaction, i.e. enzymes responsible for GA conversion: GA 20-oxidases (blue arrows), GA 3-oxidases (yellow arrows), and GA 2-oxidases (gray-green arrows).

(B) The scheme of ABA catabolic pathways in higher plants. ABA-GE, ABA-glucose ester; 7´OH-ABA, 7´-hydroxy-ABA; 8´OH-ABA, 8´-hydroxy-ABA; 9´OH-ABA, 9´-hydroxy-ABA; PA, phaseic acid; DPA, dihydrophaseic acid; neoPA, neophaseic acid. The names in grey represent minor catabolic products. In contrast to hydroxylation that leads to the irreversible ABA degradation, conjugation to glucose provides a storage form of ABA that can be converted back to bioactive ABA by glucosidases.

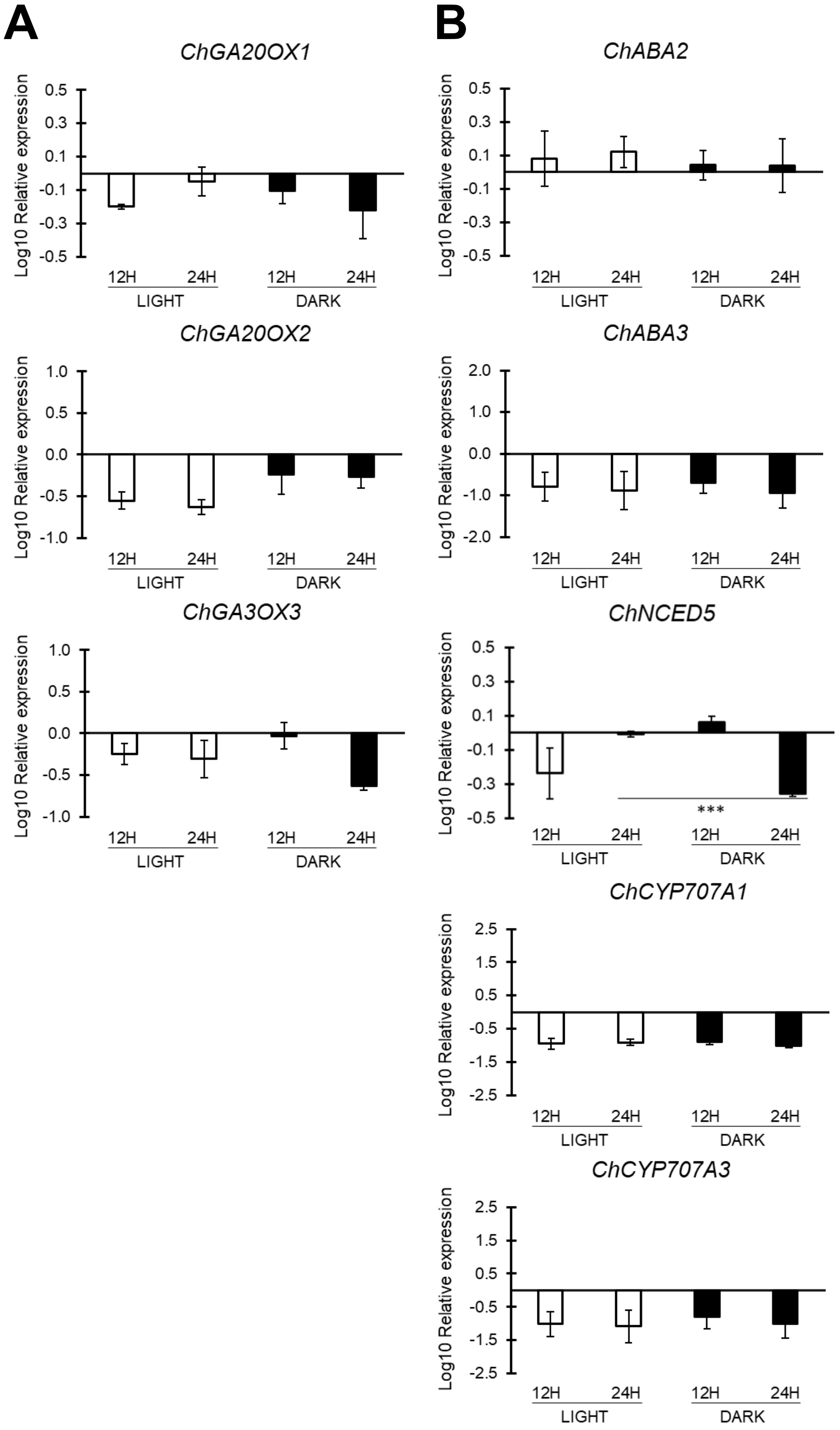

Supplemental Figure 5. Expression profiles of GA and ABA genes in wild-type seeds. **(A, B)** Relative expression level of: *ChGA20OX1*, *ChGA20OX2*, *ChGA3OX3* (A), *ChABA2*, *ChABA3*, *ChNCED5*, *ChCYP707A1, ChCYP707A3* (B) in *Cardamine* wild-type (Ox) seeds at 12 and 24 HAI (Hours After Imbibition), under light and dark conditions. Expression levels as log10 respect to the dry condition, set to 0 (X-axis). The values are means of three biological replicates, with SD values. Significant differences were analysed by t-test (***$p \leq 0.001$).

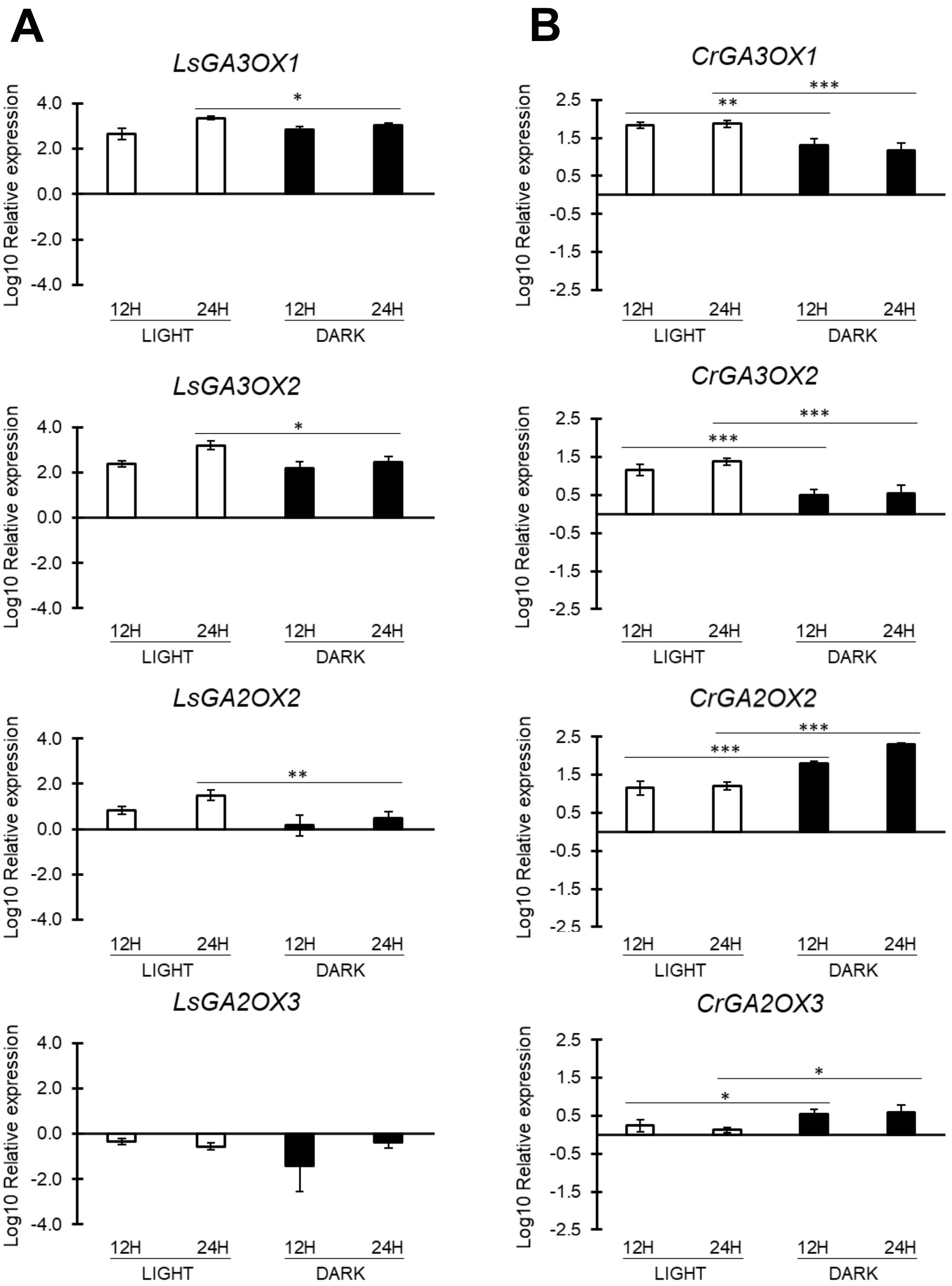

Supplemental Figure 6. Expression of GA metabolic genes in Lepidium and Capsella. (**A, B**) Relative expression level of *GA3OX1*, *GA3OX2*, *GA2OX2*, *GA2OX3* in *Lepidium sativum* (A) and *Capsella rubella* (B) wild-type seeds at 12 and 24 HAI (Hours After Imbibition), under light and dark conditions. The expression levels are presented as log10 of relative expression compared to the dry condition, which was set to 0 (indicated by the X-axis). Relative expression levels were normalized with *LsUBQ10* and *CrUBQ10* reference genes, respectively. The values of relative expression levels are means of two biological replicates, presented with SD values. Significant differences were analysed by t-test (****p* ≤0.001, ***p* ≤0.005, * *p* ≤0.05).

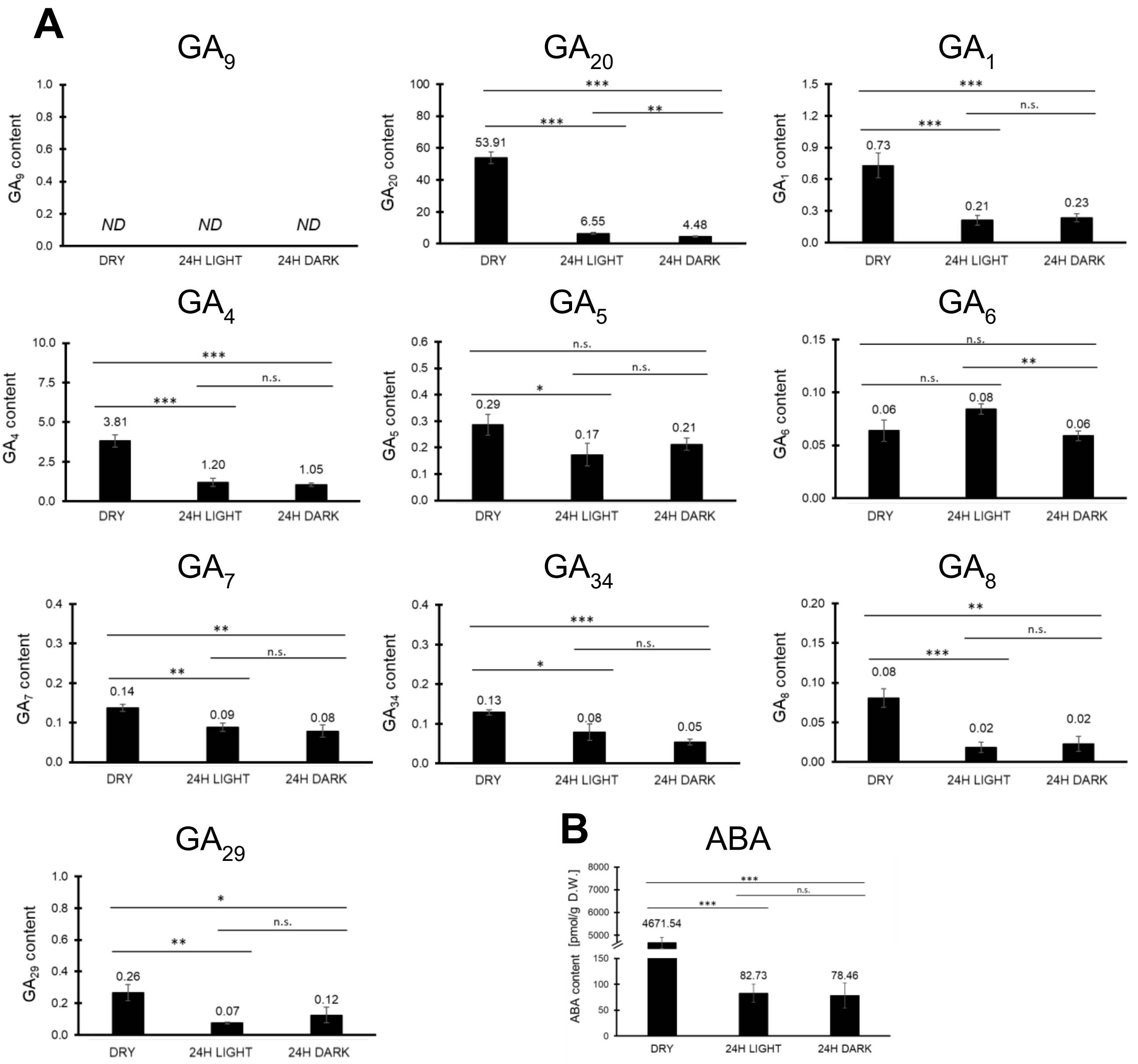

Supplemental Figure 7. GAs and ABA levels in Cardamine wild-type seeds.

(**A, B**) Hormone concentrations (pmol g–1 dry weight, DW) of precursors (GA_9 and GA_{20}), bioactive (GA1, GA_4, GA_5, GA_6, GA_7) and catabolite (GA34, GA8 and GA29) of both the 13-hydroxylated and 13-non-hydroxylated pathways (A) and of ABA (B) for Ox dry, and 24h-imbibed seeds under light and dark conditions. The values are the mean of three biological replicates, presented with SD values. Significant differences were analysed by one way ANOVA with post hoc Tukey multiple comparison test (****p* ≤0.001, ***p* ≤0.005, * *p* ≤0.05).

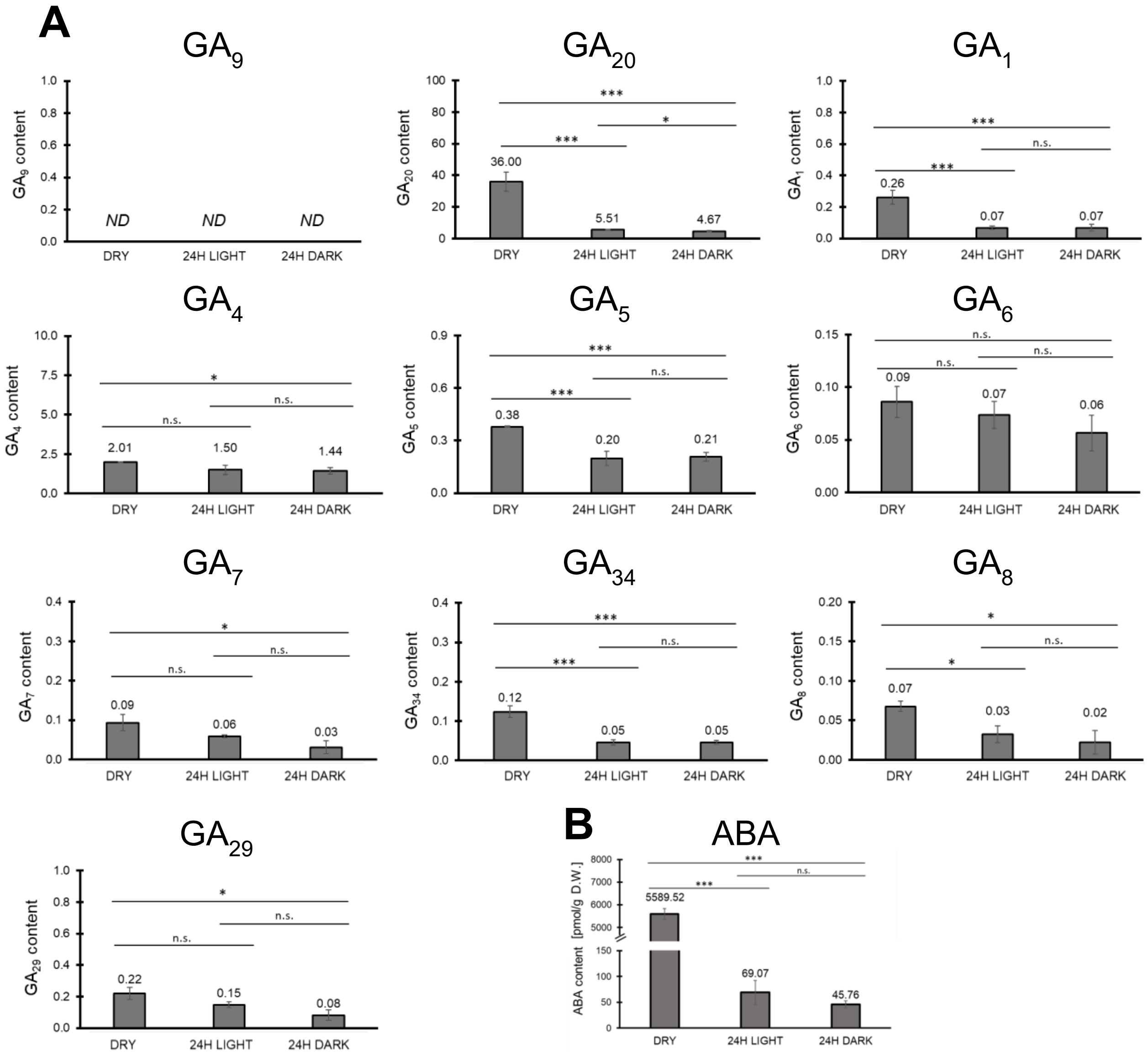

Supplemental Figure 8. GAs and ABA levels in Chdag1-1 seeds.

(**A, B**) Hormone concentrations (pmol g–1 dry weight, DW) of precursors (GA_9 and GA_{20}), bioactive (GA_1, GA_4, GA_5, GA_6, GA_7) and catabolite (GA_{34}, GA_8 and GA_{29}) of both the 13-hydroxylated and 13-non-hydroxylated pathways (A) and of ABA (B) for *Chdag1-1* dry, and 24h-imbibed seeds under light and dark conditions. The values are the mean of three biological replicates, presented with SD values. Significant differences were analysed by one way ANOVA with post hoc Tukey multiple comparison test (****p* ≤0.001, **p*<0.05).

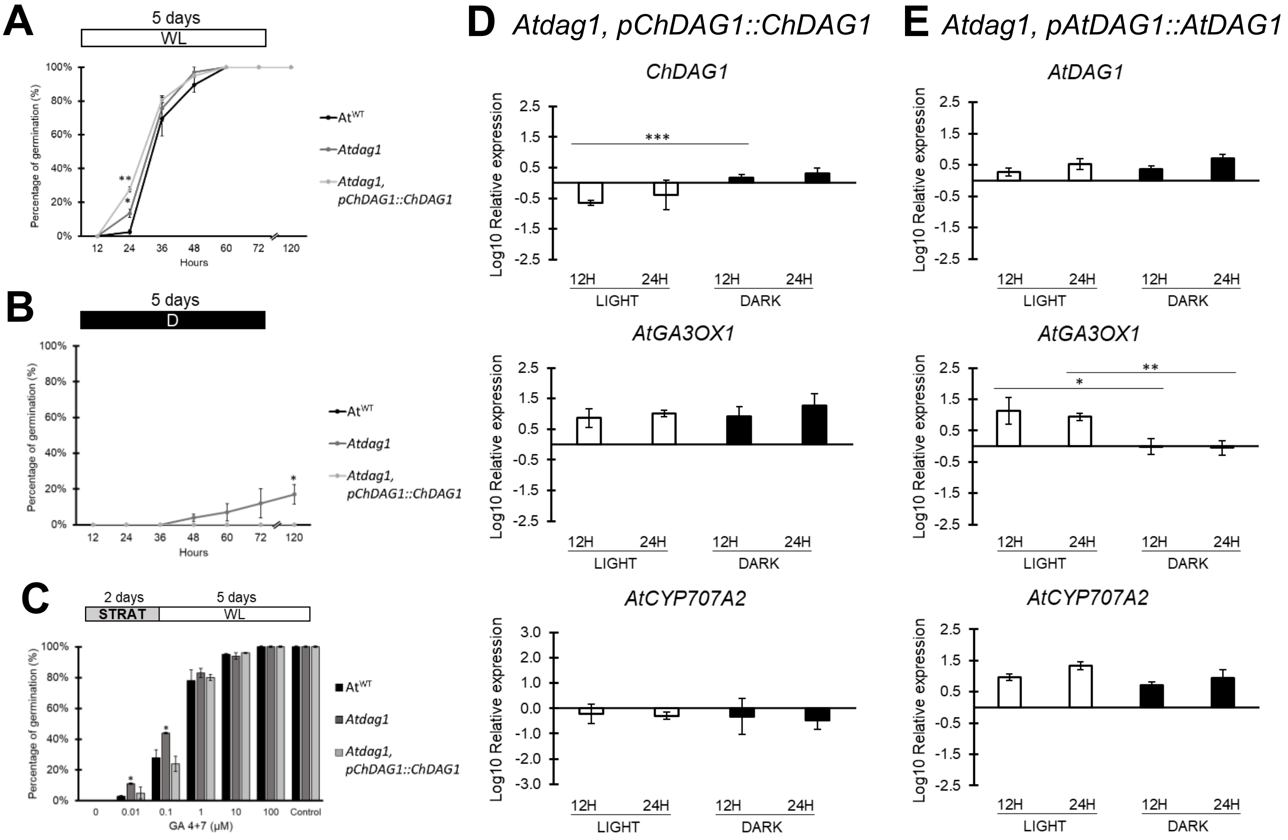

Supplemental Figure 9. Characterization of the supplemental lines of Atdag1, pChDAG1::ChDAG1 and Atdag1, pAtDAG1::AtDAG1.

(A-C) Germination rates of *Atdag1,pChDAG1::ChDAG1, Atdag1* and wild-type (WS) seeds in white light (A), in total darkness (B), and in the presence of PAC 100 µM + increasing concentrations of GA_{4+7} (C). Germination rates were measured at different HAI (12, 24, 36, 48, 60, 72, and 120) in (A and B), and at 120 HAI in (C). The values are the mean of three biological replicates, with SD values. Significant differences were analysed by t-test (***p* ≤0.005, * *p* ≤0.05). PAC: Paclobutrazol. HAI: Hours After Imbibition. Control is referred to "mock treatment control" with ethanol. The diagram on top depicts the light treatment scheme; STRAT: Stratification (2 days at 4°, Dark), WL: white light, D: dark.

(D, E) Relative expression level of *ChDAG1* and *AtDAG1* (D and E) and of *AtGA3OX1* and *AtCYP707A2* (from top to bottom). Seeds of *Atdag1,pChDAG1::ChDAG1-b* (D) and *Atdag1*, *pAtDAG1::AtDAG1-b* (E) at 12 and 24 HAI, under light and dark conditions. Expression levels as log10 respect to the dry condition, set to 0 (X-axis). The values of relative expression levels are means of three biological replicates, with SD values. Significant differences were analysed by t-test (****p* ≤0.001, ***p* ≤0.005, * *p* ≤0.05).

Atdag1, pChDAG1::ChDAG1 *Atdag1, pAtDAG1::AtDAG1*

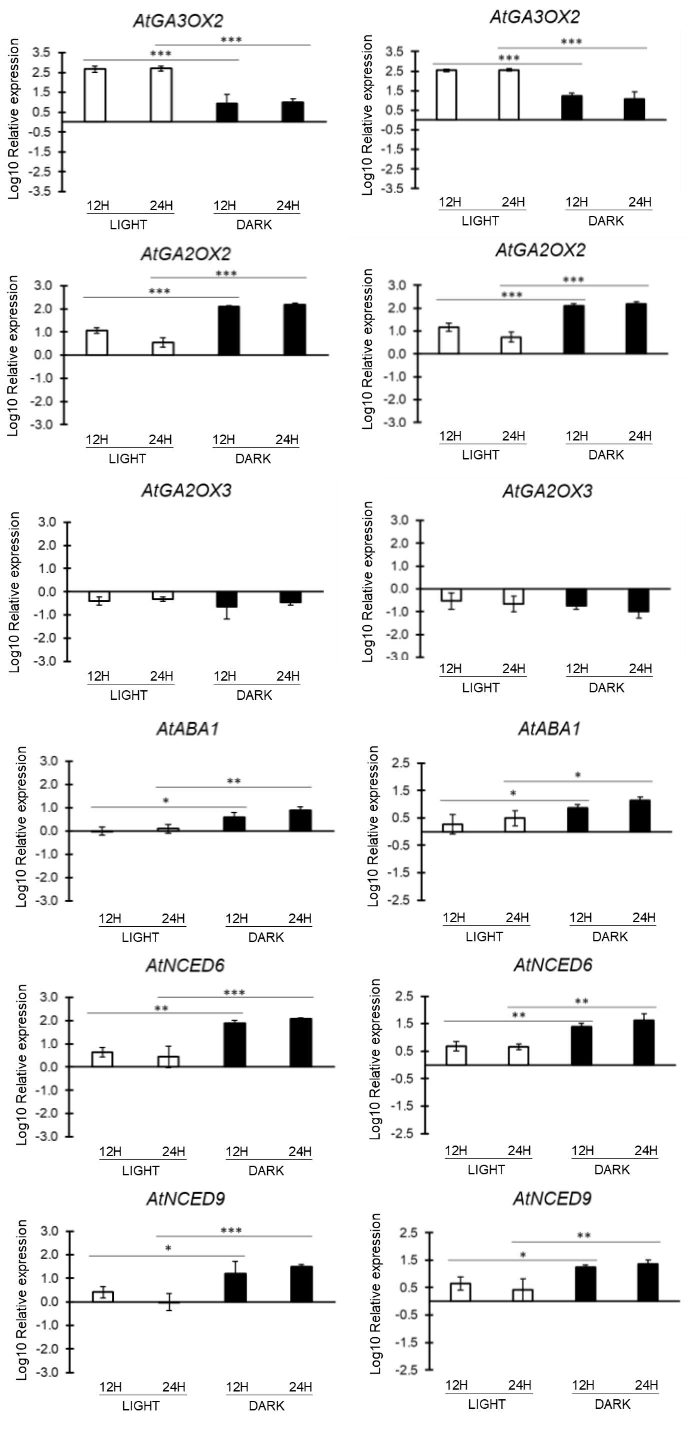

Supplemental Figure 10. Expression profiles of GA and ABA genes in Atdag1,pChDAG1::ChDAG1 and Atdag1,pAtDAG1::AtDAG1. Relative expression level of *AtGA3OX2*, *AtGA2OX2, AtGA2OX3*, *AtABA1, AtNCED6, AtNCED9* in *Atdag1,pChDAG1::ChDAG1-a* (on the left) and *Atdag1*, *pAtDAG1::AtDAG1-a* (on the right) at 12 and 24 HAI (Hours After Imbibition), under light and dark conditions. The expression levels are presented as log10 of relative expression compared to the dry condition, which was set to 0 (indicated by the X-axis). Relative expression levels were normalized with *AtUBQ10* reference gene. The values of relative expression levels are means of three biological replicates, presented with SD values. Significant differences were analysed by t-test (****p* ≤0.001, ***p* ≤0.005, * *p* ≤0.05).
