# Supplementary material for: A DOF transcriptional repressor-gibberellin feedback loop plays a crucial role in modulating light-independent seed germination
Source: Plant Commun. 2025 Jan 28;6(4):101262. doi: 10.1016/j.xplc.2025.101262 (PMC12010397; doi:10.1016/j.xplc.2025.101262)
Supplement: Table S1. Primers table [file mmc2.pdf]

Table S1 – Primers table

RT-qPCR	
Arabidopsis	
At_UBQ10_for	GGCCTTGTATAATCCCTGATGAATAAG
At_UBQ10_rev	AAAGAGATAACAGGAACGGAAACATAGT
At_DAG1_for	TGTCGAAGGTATTGGACCGAA
At_DAG1_rev	TCCGACTGGGACGTTACGA
Cardamine	
Ch_UBQ10_for	GCC AAG ATC CAG GAT AAG GA
Ch_UBQ10_rev	GGG TAG ATT CCT TCT GGA TG
Ch_DAG1_for	CGA TGG AAA CAA GAA AGG CG
Ch_DAG1_rev	ACC TTC GAC AAC CTT TGC AG
Ch_PIL5_for	CGT GTT CTG CTG TGT CAA AG
Ch_PIL5_rev	CTT CCT CAG TGG CTT CTC TT
Ch_GAI_for	TCCGCCATTTCAGATGACCT
Ch_GAI_rev	CACAAGACGACGATGAAAGG
Ch_RGA_for	CAA CCT CGC TAC TGA GAC CG
Ch_RGA_rev	CCG CGT TAG AAG ACG GTG GA
Ch_GA3OX1_for	CAA CCT GAC TTC ACA GCT CT
Ch_GA3OX1_rev	ATG AGA GGG ATG GTT TCA CC
Ch_GA3OX2_for	CCC TGG TTC ACT CGT GGT CA
Ch_GA3OX2_rev	GCC GTG GAG AAG CGA GAT CT
Ch_GA2OX2_for	ATCTGTGTGAAAGATGGAAGTTG
Ch_GA2OX2_rev	TAACACTCTTGAACCTCCCGT
Ch_GA2OX3_for	CCAAAACCCAAATCGTCAAGGC
Ch_GA2OX3_rev	TTGATGGCTTCTTGCTCCAACTG
Ch_NCED6_for	AAC CAA GCT GGG ACG ATC GG
Ch_NCED6_rev	ATC CAC GAG GCC GAT CCT AG
Ch_NCED9_for	ATG ATC TCC CAC GAA CGC CG
Ch_NCED9_rev	ATG CTG GAC TGG TTG CTC CG
Ch_CYP707A2_for	TTG GCC TTA CAT CGG AGA GA
Ch_CYP707A2_rev	TGG ACT GCT TAT CAT CAC GC
Ch_ABA1_for	TAA AAG CGG CGA CGG CTC TG
Ch_ABA1_rev	AAC CTC CGA TTC CAC CTC CG
ChGA20OX1_for	AGGCGAGAGTTGTGGCTACG
ChGA20OX1_rev	GAGCGGTTCTTCTCGTCGCT
ChGA20OX2_for	CCGAAGCTCACCGTTTCACG
ChGA20OX2_rev	AGCGTAGCCACTGCTCTCAC
ChGA20OX3_for	GTATGGCCCGACCACGACAA
ChGA20OX3_rev	CGGAAAGGAATCCGGCGAGA
ChGA3OX3_for	ACATCCCCGTGAACCGTGAC
ChGA3OX3_rev	AGGCTCGGGTTCAGGTTTGG
ChNCED5_for	ACCCGGAGACGAAGGAGCTAT
ChNCED5_rev	AACGTCCGGCGATTTCTCCC
ChABA2_for	GAGTGTTGCGGCTGAGCTTG
ChABA2_rev	CAGGCAAATGAGCCAAAGCGA
ChABA3_for	AAAGGCTCTGCGACACTCCC
ChABA3_rev	CAAGGAGAGCACCAAGCCCA
ChCYP707A1_for	GGCGGCGAAGTTTGTTCTGG

Ch CYP707A1_rev	GGCTTGTTTCCCCAGCATCC
ChCYP707A3_for	TCTCAAGCCCAGAAGCTGCG
ChCYP707A3_rev	GCATCCTCTCTTTGCTCGCC
Capsella	
Cr_UBQ10_for	GAGAGCTCCGACACCATTGACA
Cr_UBQ10_rev	ACGCTGCTGGTCCGGAGG
Cr_GA3OX1_for	GACCGAGCTATGGGTCTAGC
Cr_GA3OX1_rev	ACCGGCGGTATTGTTCTGGT
Cr_GA3OX2_for	ATGGCTTACCTTTGGGGTCCA
Cr_GA3OX2_rev	GCAAGGTACTGTTTCCAAGTGAG
Cr_GA2OX2_for	TGGGGAACACACAGACCCAC
Cr_GA2OX2_rev	AGGGACAGCGACCCAACTTC
Cr_GA2OX3_for	CGACCCGATCTTTTGACCCG
Cr_GA2OX3_rev	GGTGGACCCGCTTTGTCTTT
Lepidium	
Ls_UBQ10_for	GAGAGCTCCGACACCATTGACA
Ls_UBQ10_rev	ACGCTGCTGGTCCGGAGG
Ls_GA3OX1_for	GACCGAGCTATGGGTCTAGC
Ls_GA3OX1_rev	ACCGGCGGTATTGTTCTGGT
Ls_GA3OX2_for	ATGGCTTACCTTTGGGGTCCA
Ls_GA3OX2_rev	GCAAGGTACTGTTTCCAAGTGAG
Ls_GA2OX2_for	TGGGGAACACACAGACCCAC
Ls_GA2OX2_rev	AGGGACAGCGACCCAACTTC
Ls_GA2OX3_for	AGTTCTTTGCTCTGCCTCGC
Ls_GA2OX3_rev	GCCCGATCCGTTTACTACCG
GUS	
pChDAG1::GUS_fw	AAGCTTTCTAGGACGTGATCTTTT
pChDAG1::GUS_rev	TCTCTTTGTCCAAGTTCGGATCC
Transgenic plants	
pAtDAG1_for	AGTTTCGAATTCTGGTGGCTA
pAtDAG1_rev	GCCTTTCTCTTTGTTGAAGTTC
geneAtDAG1_for	ATGGATGCTACGAAGTGGACTCAG
geneAtDAG1_rev	CAG GAG GAT CTT CAT GGT GA
pChDAG1_for	CAT ATG TAC ATA CCT TCT TAA GCC C
pChDAG1_rev	CTC TGC CTT TCT CTT TGT CCA AGT
geneChDAG1_for	ATGGATGCTACGAAGTGGACGCAG
geneChDAG1_rev	TAATACAGGAGGATCCTCGTGGTAA
